# Dopamine Neurons Change the Type of Excitability in Response to Stimuli

**DOI:** 10.1371/journal.pcbi.1005233

**Published:** 2016-12-08

**Authors:** Ekaterina O. Morozova, Denis Zakharov, Boris S. Gutkin, Christopher C. Lapish, Alexey Kuznetsov

**Affiliations:** 1 Department of Physics, Indiana University, Bloomington, Indiana, United States of America; 2 Department of Mathematical sciences, Indiana University - Purdue University, Indianapolis, Indiana, United States of America; 3 Institute of Applied Physics, Nizhny Novgorod, Russia; 4 Group of Neural Theory, INSERM U960 LNC, IEC, Ecole Normale Superieure PSL University, Paris; 5 Center for Cognition and Decision Making, NRU HSE, Moscow, Russia; 6 Addiction Neuroscience Program, Indiana University - Purdue University, Indianapolis, Indiana, United States of America; George Mason University, UNITED STATES

## Abstract

The dynamics of neuronal excitability determine the neuron’s response to stimuli, its synchronization and resonance properties and, ultimately, the computations it performs in the brain. We investigated the dynamical mechanisms underlying the excitability type of dopamine (DA) neurons, using a conductance-based biophysical model, and its regulation by intrinsic and synaptic currents. Calibrating the model to reproduce low frequency tonic firing results in *N*-methyl-D-aspartate (NMDA) excitation balanced by γ-Aminobutyric acid (GABA)-mediated inhibition and leads to type I excitable behavior characterized by a continuous decrease in firing frequency in response to hyperpolarizing currents. Furthermore, we analyzed how excitability type of the DA neuron model is influenced by changes in the intrinsic current composition. A subthreshold sodium current is necessary for a continuous frequency decrease during application of a negative current, and the low-frequency “balanced” state during simultaneous activation of NMDA and GABA receptors. Blocking this current switches the neuron to type II characterized by the abrupt onset of repetitive firing. Enhancing the anomalous rectifier Ih current also switches the excitability to type II. Key characteristics of synaptic conductances that may be observed in vivo also change the type of excitability: a depolarized γ-Aminobutyric acid receptor (GABAR) reversal potential or co-activation of α-amino-3-hydroxy-5-methyl-4-isoxazolepropionic acid receptors (AMPARs) leads to an abrupt frequency drop to zero, which is typical for type II excitability. Coactivation of *N*-methyl-D-aspartate receptors (NMDARs) together with AMPARs and GABARs shifts the type I/II boundary toward more hyperpolarized GABAR reversal potentials. To better understand how altering each of the aforementioned currents leads to changes in excitability profile of DA neuron, we provide a thorough dynamical analysis. Collectively, these results imply that type I excitability in dopamine neurons might be important for low firing rates and fine-tuning basal dopamine levels, while switching excitability to type II during NMDAR and AMPAR activation may facilitate a transient increase in dopamine concentration, as type II neurons are more amenable to synchronization by mutual excitation.

## Author Summary

Dopamine neurons play a central role in guiding motivated behaviors. However, complete understanding of computations these neurons perform to encode rewarding and salient stimuli is still forthcoming. Network connectivity influences neural responses to stimuli, but so do intrinsic excitability properties of individual neurons, as such properties define synchronization qualities and neural coding strategy. We investigated the excitability type of the DA neuron and found that, depending on the synaptic and intrinsic current composition, DA neurons can switch from type I to type II excitability. In short, without synaptic inputs or under balanced excitatory and inhibitory inputs DA neurons exhibits type I excitability, while excitatory AMPAR inputs can switch the neuron to type II. Type I neurons are best suited for coding the stimulus intensity due to their ability to decrease the firing rate smoothly. Type I excitability might be important for achieving low a basal DA concentration necessary for normal brain functioning. Switching to type II excitability further enables robust transient DA release of heterogeneous DA neuron population in response to correlated inputs, partially due to evoked population synchrony.

## Introduction

Midbrain dopamine (DA) neurons predominantly fire in a low frequency, metronomic manner (i.e. tonic) and display occasional, yet functionally important, high frequency, burst-like episodes [1,2]. While regular tonic firing is observed in isolated preparations (i.e. slices), tonic firing pattern in vivo is somewhat more variable due to active synaptic inputs [3,4]. Such tonic activity is important for maintaining a constant basal level of dopamine in projection areas. Accordingly, abnormal basal DA levels are linked to psychiatric disorders from depression to schizophrenia [5,6]. While the maintenance of basal DA levels seem to be critical for normal brain function, a consistent picture has not yet emerged regarding how changes in firing patterns of the DA neuron facilitates this important biological function.

Background activity of the DA neuron appears to rely on the intrinsic pacemaking mechanism that generates tonic firing. The current composition producing low-frequency pacemaking in DA neurons is of vibrant debate among researchers in the field. A number of experimental [7–19] studies suggests that the maintenance of tonic firing in at least a subpopulation of DA neurons relies on the interactions of the voltage gated calcium (Ca^2+^) and SK-type Ca^2+^-dependent potassium (K^+^) currents Slow pacemaking in our model relies on a subthreshold Ca^2+^-K^+^ oscillatory mechanism, similar to a number of well-established models [4,20–24]. Interaction between Ca^2+^ and Ca^2+^-dependent K^+^ currents periodically brings the neuron to the spike threshold and generates a metronomic firing pattern. In our model, spike-producing currents (fast sodium and the delayed rectifier potassium) play a mostly subordinate role in this dynamic, adding a spike on top of the oscillations without significant changes to the period or shape of voltage and calcium oscillations, as in the study by Wilson and Callaway 2000 [4]. A number of studies suggest an additional Ca^2+^-independent oscillatory mechanism [25–27]. In particular, they emphasize the contribution of sodium currents to pacemaking. We review the literature on the mechanisms of DA neuron pacemaking in the discussion section in more detail. The specific composition of currents contributing to oscillations determines the response of the DA neurons to stimuli, their synchronization properties and, ultimately, the computations they perform. In this paper, we use recent experiments to calibrate the dynamical properties of the DA neuron and determine its excitability type.

A standard method to classify neuronal excitability is via characterizing the frequency-to-input relationship, or F-I curve. Two major types of excitability can be determined based on how the onset of tonic firing occurs as the applied current increases and the neuron is released from quiescence at the hyperpolarized rest state [28]. A type I-excitable neuron can fire at an arbitrary low frequency near the onset of firing, whereas a type II neuron shows a discontinuous jump to a minimal frequency above a certain current threshold and fires only in a limited range of frequencies [21]. Generally, the onset of repetitive firing occurs through one of two mechanisms: 1) a saddle-node bifurcation on invariant circle or SNIC (type I excitability) or 2) an Andronov-Hopf bifurcation (type II excitability) [29]. Type II neurons, such as fast-spiking inhibitory interneurons in the cortex, display precise spike timing even in the presence of noise and are therefore suitable for the implementation of spike time coding [30,31]. A type I neuron, such as a weakly adapting cortical pyramidal neuron, was shown to relay the stimulus rate by modulating its own frequency, and, therefore, displayed rate coding [31]. Further, type II neurons display resonance and controlled synchronization in networks [31–33]. For neurons that are tonically active without any injected current, such as DA neurons, the transition to the non-spiking rest state occurs when a sufficiently strong hyperpolarizing current is injected. For these neurons, the excitability type would be defined by the transition from tonically firing to quiescent/excitable: again type I would show a smooth frequency decrease to zero, while type II should show an abrupt transition to quiescence. At this point, there is no direct evidence defining to what type of excitability DA neurons belong, and how the different intrinsic conductances and the different synaptic inputs influence their type. Determining the type of excitability will allow us to predict the behavior of the DA neuron during application/blockade of different currents and better understand computations it performs in different input conditions (e.g. rate coding vs. resonance at a particular input frequency).

A number of experimental studies provide indirect evidence of excitability type of the neuron in control conditions and during activation of synaptic inputs. It has been shown that the firing rate of DA neurons increases linearly in response to a ramping depolarizing current until it goes into depolarization block (e.g. [3,34]). Further, injection of a tonic hyperpolarizing current to the regularly firing DA neuron in vitro increases its interspike intervals [7]. The firing properties of the neuron in response to a combination of tonic inhibitory and excitatory synaptic conductances were investigated by Lobb and colleagues [35,36]. Using the dynamic clamp technique, they injected inhibitory γ-Aminobutyric acid (GABA) and excitatory *N*-methyl-D-aspartate (NMDA) receptor conductances in SNc DA neurons. Injection of tonic GABAR conductance decreased the firing rate of the neuron several-fold. Furthermore, the neuron fired at low frequencies when NMDAR and GABAR conductances balanced each other. Thus, NMDAR activation, which strongly increases the firing frequency [37–40], can be effectively compensated by GABAR activation. Such compensation would be impossible if the inhibition produced an abrupt transition to quiescence and the neuron jumped from a high frequency to zero. The experimentally observed compensation suggests, again, a smooth frequency decrease upon GABAR activation rather than an abrupt transition to the resting state at hyperpolarized potentials. Together, these data resemble the tonic firing/quiescence transition in type I neurons with two distinctions. First, the transition parameter is not an injected current, but an ohmic GABAR conductance. In experiments, a conductance has already been used instead of an injected current to determine the neuronal excitability [41]. Second, the co-activation of the NMDA receptor introduces an additional parameter (its maximal conductance). Both of these extend the definition of excitability into the space of synaptic conductances. Formally, the excitability type is an intrinsic property of a neuron, yet viewing synaptic inputs as changing excitability of a neuron is a powerful concept used to understand neuron dynamics *in vivo* [31,42]. We used the experiments described above to parameterize a model of the DA neuron, determine its type of excitability, and determine how intrinsic and synaptic currents shape the excitability type and, therefore, the computational properties of the neuron.

These experiments suggest that the DA neuron exhibits type I excitability in isolation from synaptic inputs and under the balanced influence of excitatory and inhibitory synaptic conductances. However, the excitability type has been shown to vary depending on the intrinsic currents and network connectivity [42,43]. For example, modeling results suggest that changes in the intrinsic currents, e.g. L-type Ca^2+^ current, can switch the excitability type of the DA neuron [44,45]. Here we address the variability in the excitability type under different conditions by studying the contribution of intrinsic and synaptic currents to regulation of the low-frequency DA neuron firing.

## Results

### Compensatory action of asynchronous NMDA and GABA inputs

**Fig 1:**
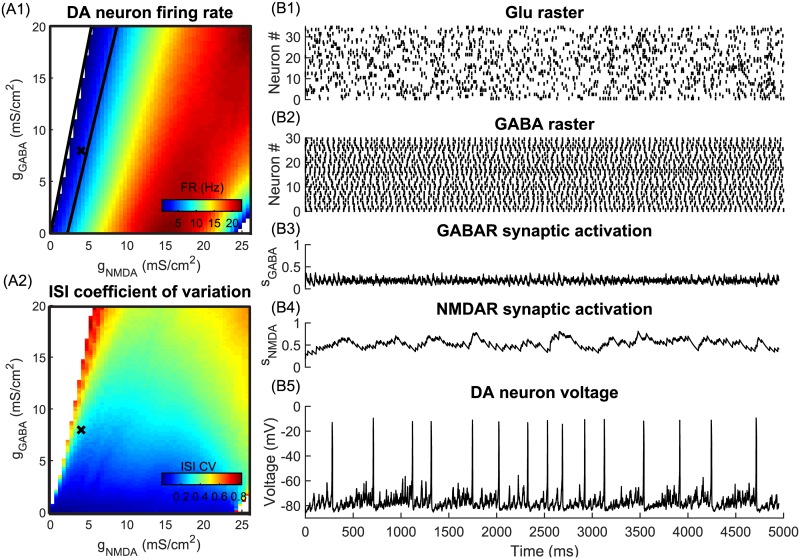
The rate and regularity of the DA neuron firing receiving asynchronous synaptic Glu and GABA inputs. (A1) The firing rate. Balanced activity of Glu and GABA populations results in low-frequency DA neuron firing (between black lines) (A2) Coefficient of variation (CV) of the ISI. For majority of *g*_*NMDA*_, *g*_*GABA*_ values, CV<0.5, indicating low variability in DA neuron firing. (B 1–5) Balanced asynchronous GABA and Glu inputs provide constant noisy levels of inhibition and excitation respectively and result in DA firing rates similar to the background firing rates.

We investigated the behavior of a simulated dopamine neuron in response to irregular asynchronous GABA and glutamate (Glu) inputs to mimic temporal structure of neural firing in *in vivo* conditions. The Glu input was produced by Poisson distributed spike trains and GABA inputs was explicitly modeled as activity of a population of GABA neurons (detailed description of the inputs and equations are given in the methods section). We quantified changes in the firing rate and the regularity of DA neuron firing in response to synaptic inputs of different strengths (Fig 1A1 and 1A2). We identified a parameter region where the excitatory and inhibitory inputs balance to produce low frequency DA neuron firing at rates similar to background firing (Fig 1A1, between the black lines). This happens because asynchronous GABA and Glu inputs (see rasters in Fig 1B1 and 1B2) activate GABARs and NMDARs nearly tonically (Fig 1B3 and 1B4) and provide quasi-constant levels of inhibition and excitation to the DA neuron respectively. Under the influence of these two inputs, the DA neuron fires similarly to the *in vitro-*like conditions (tonic inputs), but with less regularity, which is typical of the background firing *in vivo*. An example voltage trace of the DA neuron in response to synaptic inputs formed by asynchronous Glu and GABA populations is shown in Fig 1B5. Fluctuations in the firing of neural populations innervating the DA neuron can produce irregular spiking as observed in *in vivo* experiments.

Considering that asynchronous inputs produce nearly constant receptor activation (see Fig 1B3 and 1B4), for further analysis we substituted these Glu and GABA inputs by tonic currents. Moreover, tonic synaptic currents mimic long-lasting injection of the conductances in dynamic clamp experiments [46,35,36], or iontophoresis of the agonists [39,40], or bath application of the agonists. Transition from asynchronous inputs to tonic currents is described in the methods section.

### Balance of tonic NMDA and GABA inputs

**Fig 2:**
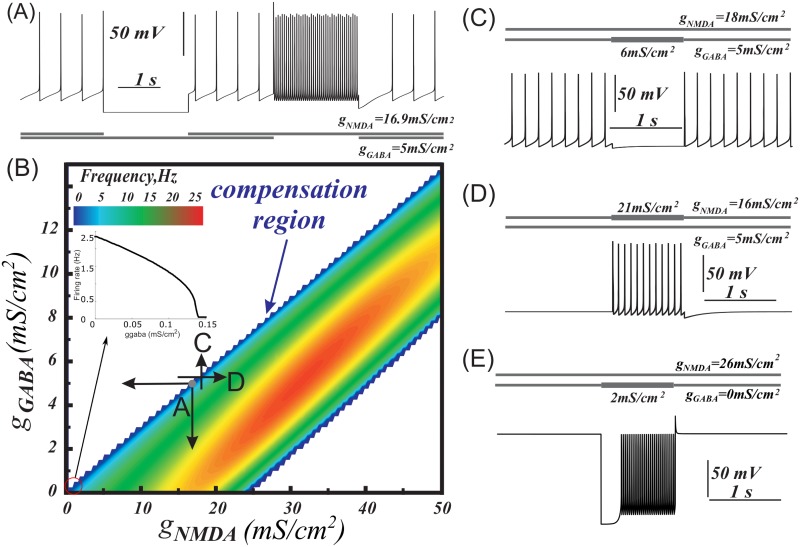
Balance of NMDA and GABA receptor activation and two ways of eliciting bursts or pauses of the DA neuron. (A) Tonic co-activation of NMDA and GABA receptors balances each other and supports low frequency firing in the DA neuron model. Transient deactivation of NMDAR produces a pause, whereas deactivation of GABAR results in a high-frequency burst. (B) Heat plot of the frequency distribution on the plane of NMDAR and GABAR conductances. The insert illustrates a smooth frequency decrease during application of GABAR conductance (*g*_*NMDA*_ = 0 mS/cm^2^). (C) As an alternative to the deactivation of NMDAR in (A), a pause may be produced by further activation of GABAR. (D) Strengthening NMDA excitation produces a burst, even if GABAR activation initially blocks firing. (E) Activation of GABAR can rescue DA neuron from depolarization block. To match the experimental conditions in Lobb at al. (2010) [50], we set α-amino-3-hydroxy-5-methyl-4-isoxazolepropionic acid receptor (AMPAR) current to 0.

Our next goal was to reproduce the experimentally-observed compensatory influence of tonic NMDAR and GABAR conductances [35]. Using the dynamic clamp technique, it was shown *in vitro* that a balanced injection of GABAR and NMDAR conductances leads to DA neurons firing at frequencies comparable with background frequencies (1–5 Hz). Removal of inhibition in such conditions evokes a classical disinhibition burst (the disinhibition model of burst generation is well known and described in e.g. [35,47–49]). Fig 2A reproduces the voltage traces obtained in the experiments by Lobb et al. 2010 [35]. In this example, the simulated DA neuron is tonically active at 1.5 Hz during tonic co-activation of NMDA and GABA receptors (*g*_*NMDA*_ = 16.9 m S/cm^2^, *g*_*GABA*_ = 5*mS*/cm^2^). Removal of the GABAR conductance produces an episode of high-frequency firing (Fig 2A). Removal of the NMDAR conductance produces a pause in firing (Fig 2A).

We explored the range of NMDAR and GABAR conductances that produce tonic firing in the DA neuron model (Fig 2B). Compensation of NMDAR and GABAR activation can be readily achieved near the upper boundary of the firing region (Fig 2B, blue). When both receptors are activated, low frequency tonic activity is observed (Fig 2A). The dot labeled as A on the heat plot indicates conductances taken for this simulation. As in the experiments [35], the balanced region is stretched linearly on the conductance plane with NMDA/GABA slope around 3.4. Moving to the left on the diagram corresponds to deactivation of the NMDAR current and blocks DA neuron firing due to the remaining GABAR activation (Fig 2A). A pause may also be produced by stronger activation of the GABAR (Fig 2C). Conversely, moving down on the diagram corresponds to deactivation of the GABAR and evokes high-frequency firing (Fig 2A). The firing frequency also increases by moving from the upper boundary of the firing region to the right (increasing NMDAR conductance; Fig 2D). These two directions correspond to two ways of eliciting a DA neuron burst: strengthening NMDA excitation or removing inhibition, respectively.

Excessive tonic NMDAR activation leads to a depolarization block, as shown in Fig 2B and 2E at high NMDA and low GABA receptor conductances. Interestingly, application of a tonic GABAR conductance in combination with an excessive NMDAR conductance may rescue high-frequency firing in the model (Fig 2E). Thus, the compensatory influence of GABAR activation removes depolarization block induced by an excessive NMDAR activation and restores the intrinsic oscillatory mechanism required for tonic firing.

### DA neuron excitability under control and during balanced input conditions: Type I

The smooth frequency decrease to zero as the neuron transition to quiescence when GABAR conductance increases suggests type I excitability for the DA neuron both in *in vivo* and *in vitro* like cases (Figs 1A1 and 2B). However, excitability is classically defined by the structure of the transition between spiking and hyperpolarized rest state induced by an injected current, as opposed to a synaptic conductance. We show that the DA neuron exhibits type I excitability by standard definition with a continuous F-I curve and place it in Supporting Information (S1 Fig) as this case has less physiological significance than the influence of synaptic currents. We further investigate the influence of intrinsic and synaptic currents on the excitability type of the DA neuron.

### Influence of intrinsic currents on the type of excitability

#### The role of Ca^2+^ and Ca^2+^-dependent K^+^ currents

The subthreshold Ca^2+^-K^+^ oscillatory mechanism underlies the generation of low frequency background firing in a significant subpopulation of DA neurons [7–18,51]. However, a number of studies suggest a contribution of Ca^2+^-independent currents to oscillations [26,19,25,27,46]. In accord with these studies, we found that, if considered in isolation, the Ca^2+^-K^+^ oscillatory mechanism provides type II excitability, which is incompatible with the experiments reproduced above. In order to study this, we block the subthreshold sodium current to isolate only the Ca^2+^- and Ca^2+^-dependent K^+^ currents that constitute the oscillatory mechanism. The reduced model is described by the following system of equations:

$$\begin{array}{l} c_{m}\frac{dv}{dt}=\overset{I_{Ca}}{\overset{︷}{g_{Ca}(v)(E_{Ca}-v)}}+\overset{I_{KCa}+I_{K}}{\overset{︷}{\left(g_{KCa}([Ca^{2+}])+g_{K}(v))(E_{K}-v\right)}}+\overset{I_{leak}}{\overset{︷}{g_{l}(E_{l}-v)}},\\ \frac{d[Ca^{2+}]}{dt}=\frac{2\beta }{r}\left(\frac{g_{Ca}(v)}{zF}(E_{Ca}-v)-P_{Ca}[Ca^{2+}]\right) \end{array}$$

**Fig 3:**
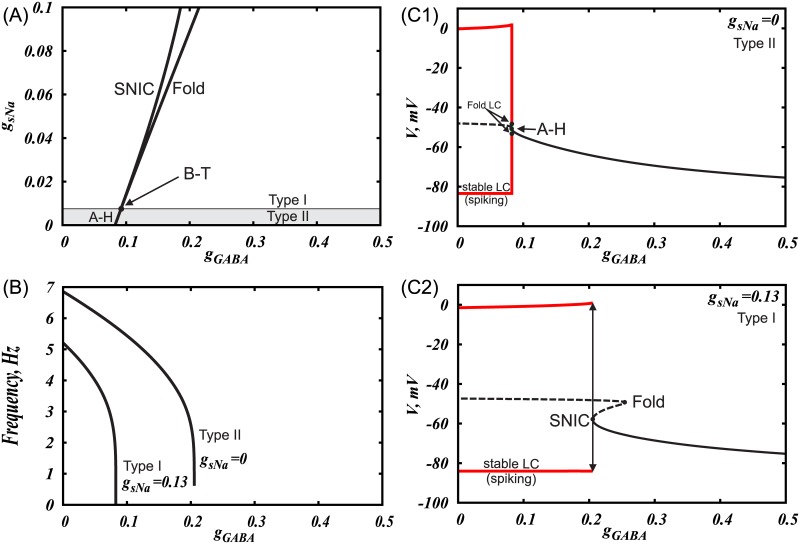
Application of GABA leads to termination of oscillations through Andronov-Hopf bifurcation in a DA neuron, which pacemaking produced by pure Ca^2+^-K^+^ oscillatory mechanism. (A) Two-parameter bifurcation diagram showing transition from type II to type I excitability produced by increasing subthreshold sodium current. (B) F-I curves for two cases shown in (C1) and (C2). Subthreshold sodium current enables smooth frequency decrease to zero upon GABAR activation. One-parameter bifurcation diagrams in cases when subthreshold sodium current is absent (C1) and present (C2). SNIC stands for a saddle node bifurcation on invariant circle, B-T is Bogdanov-Takens bifurcation, A-H is Andronov–Hopf bifurcation, LC stands for a limit cycle.

The interaction of the L-type voltage-gated Ca^2+^ and the calcium-dependent potassium currents periodically brings the neuron to the spike threshold and generates a metronomic firing activity pattern. Application of an inhibitory input (GABA) to this model reduces the amplitude of voltage oscillations instead of decreasing the firing frequency gradually (Fig 3). This transition is typical for systems where the oscillations are terminated via an Andronov-Hopf bifurcation: the oscillatory trajectory (limit cycle) decreases in amplitude and merges with an equilibrium state. An abrupt transition to zero frequency quiescence upon hyperpolarization suggests a type II excitability of the neuron. Thus, these currents are important for producing metronomic firing, but other intrinsic currents allow for a gradual frequency decrease during application of a hyperpolarizing input observed experimentally.

#### The role of a subthreshold sodium current

**Fig 4:**
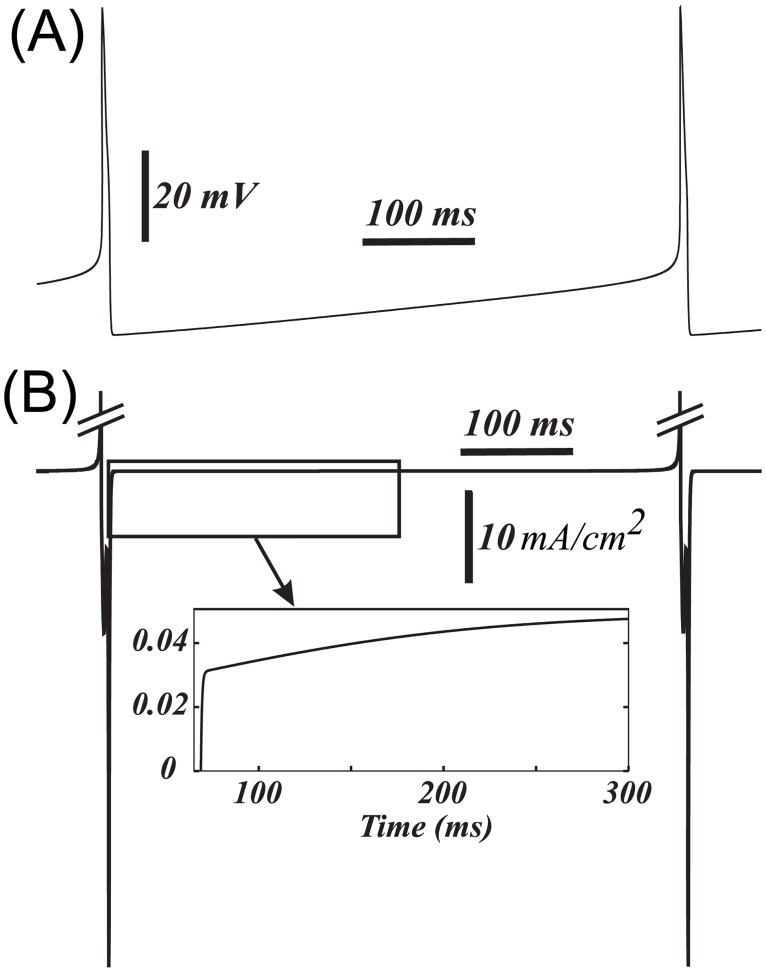
The time series of the total ionic current (B) shows near cancellation of the current during the interspike interval, which creates a bottleneck effect. The voltage is shown in (A) for a reference.

We found that the subthreshold sodium current is necessary for gradual frequency decreases during application of a hyperpolarizing input, as well for a frequency range that spans the observed control frequencies of the DA neurons during a balanced tonic NMDAR and GABAR activation. The reduced firing frequency in the balanced state comes about because the inputs create a slow “bottleneck” effect in the subthreshold voltage range, where the hyperpolarizing inputs nearly cancel the depolarizing ones (Fig 4, also see methods, *subthreshold currents* section for a more detailed description) and expand the interspike interval. This input balance is achieved due to the contribution of the subthreshold sodium current into the pacemaking mechanism of the DA neuron. By contrast, in the reduced model that includes only Ca^2+^ and Ca^2+^-dependent K^+^ currents into the mechanism, the inhibitory input cannot restore appropriate frequency, but instead blocks the voltage oscillations. The inclusion of the subthreshold sodium current allows the firing frequency to vary without compromising the oscillatory mechanism. It allows us to reduce the frequency to an arbitrary low value upon application of a hyperpolarizing input and thus leads to type I excitability of the DA neuron.

#### A mechanism for NMDA-GABA balance: SNIC bifurcation

**Fig 5:**
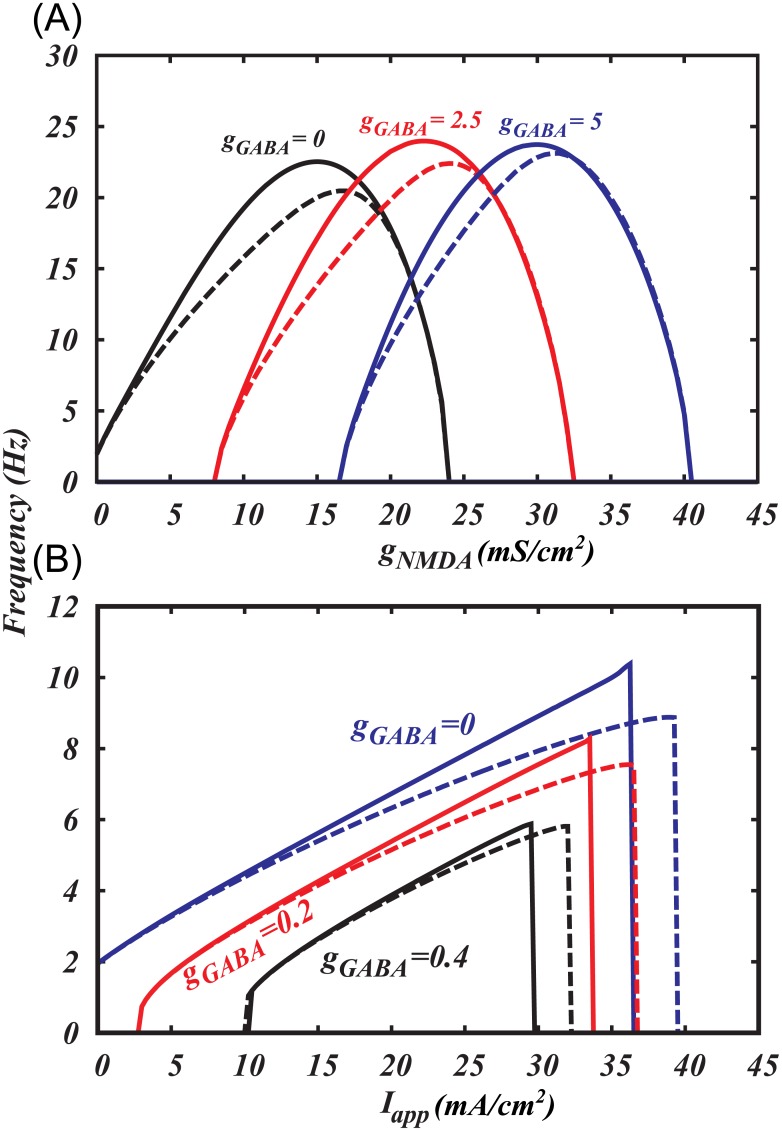
Spike-producing currents (I_Na_ and I_DR_) have no significant influence on the frequency growth induced by NMDA receptor activation (A) or an injected current (B). The frequencies are measured in the model with (dashed) and without (solid) these currents.

Mathematical analysis allows for a better explanation of how the subthreshold sodium current changes the response of the neuron to a combination of excitatory and inhibitory inputs. First, we further reduce the model by separating the slow dynamics from the fast dynamics and removing the fast sodium and the delayed rectifier potassium spike-producing currents. We now define the model as having fired a spike when the voltage crosses a putative spike-threshold (set at -40 mV). Fig 5 shows that the frequencies displayed by the model do not significantly change. This reproduces the experiments, in which blockade of the spike-producing currents do not significantly change either the frequency of background oscillations [4] or the NMDA-evoked high frequencies [40]. Additionally, model behavior with fast sodium current and when it is blocked is shown in S2 Fig. It illustrates that full DA neuron is type I excitable as well as the reduced model (without spike-producing currents, but with the subthreshold Na^+^ current). The reduction to the slow-dynamics subsystem decreases the number of variables in the model and enables standard nullcline analysis because it applies only to two-dimensional systems [29,52] (see methods for the description of the nullcline analysis).

**Fig 6:**
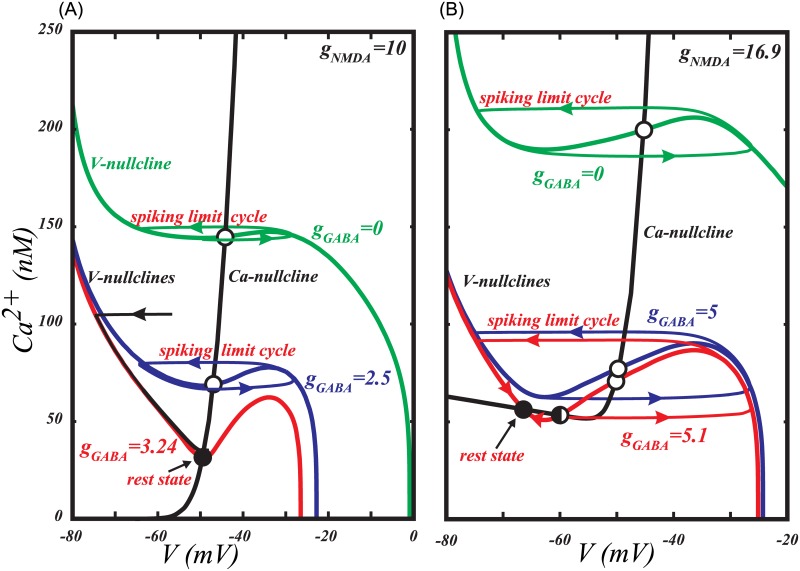
The subthreshold sodium current changes the type of transition to hyperpolarized rest state induced by GABAR activation. In both cases the growing GABAR conductance leads to a downward shift of the voltage nullcline (solid folded curve). (A) In the pure Ca^2+^-K^+^ mechanism for voltage oscillations, inhibition leads to a transition to the rest state through an Andronov-Hopf bifurcation, which occurs with little change in the firing frequency. The Andronov-Hopf bifurcation is defined as the disappearance of a closed trajectory representing firing (limit cycle) by shrinking in amplitude and merging with an equilibrium state. (B) If the Ca^2+^-K^+^ mechanism is augmented by a subthreshold Na^+^ current, the transition occurs through a Saddle-Node on Invariant Circle bifurcation (SNIC), which corresponds to a gradual decrease in the frequency to zero. The SNIC bifurcation is defined as the emergence of new equilibrium states that interrupt the limit cycle.

Our reduced Ca^2+^-K^+^ model for voltage oscillations (e.g. [4]) shows that the transition to quiescence at a hyperpolarized voltage occurs via an Andronov-Hopf bifurcation (Fig 3), in which oscillations disappear without a decrease in the frequency. Fig 6 presents standard nullcline analysis of the model, which explains oscillatory behavior and bifurcations mechanistically. In Fig 6A, the Andronov-Hopf bifurcation occurs as the voltage nullcline shifts down and simultaneously to the right, so that its intersection with the Ca^2+^ nullcline moves across the minimum. In the model with the subthreshold sodium current and Ca^2+^ leak current (Fig 6B), the minimum of the voltage nullcline is further away from the steep part of the Ca^2+^ nullcline, so that, when the voltage nullcline shifts down, its minimum touches the flat part of the Ca^2+^ nullcline. The proximity of the minimum of the voltage nullcline and the bottom part of the Ca^2+^ nullcline creates a “bottleneck” effect: The closer the nullclines, the smaller the vector field (the rate of change) in this neighborhood. The limit cycle is channeled through the gap between the nullclines and, accordingly, the oscillation evolves slowly. In the limiting case, when the minimum of the voltage nullcline touches the bottom part of the Ca^2+^ nullcline, a saddle-node on invariant circle (SNIC) bifurcation occurs: two equilibrium states, a stable (node) and an unstable (saddle) emerge, interrupt the limit cycle, and the period becomes infinite. The closer the bifurcation parameter *g*_*GABA*_ to the bifurcation value, the more time the voltage spends in the bottleneck, creating a long interspike interval. Thus, by introducing the subthreshold sodium current, we change the bifurcation that leads to the quiescence at hyperpolarized potentials from Andronov-Hopf to SNIC.

#### The role of Ih current

**Fig 7:**
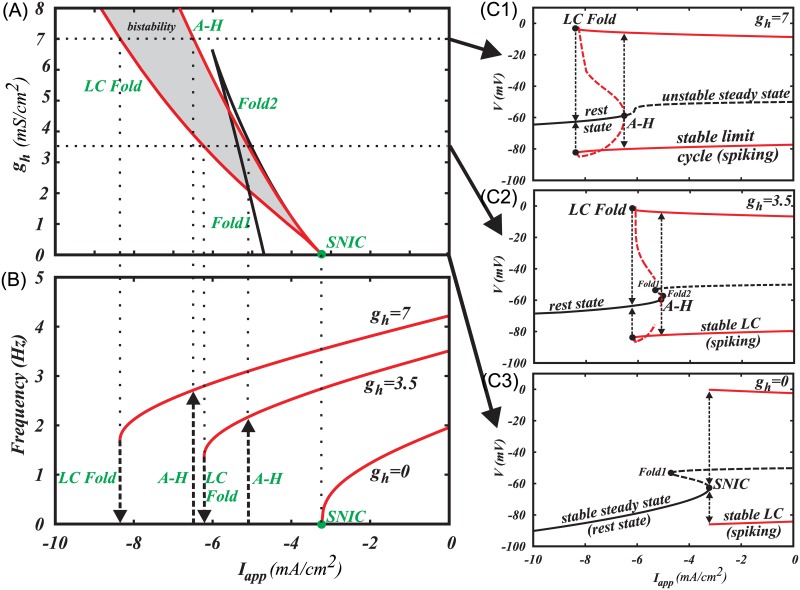
The Ih current switches the type of excitability of the DA neuron to type II. (A) Two-parameter bifurcation diagram. See part (C) for explanations. LC Fold is a saddle-node bifurcation of limit cycles, in which two oscillatory solutions, stable and unstable, emerge. A-H is the Andronov-Hopf bifurcation, in which a limit cycle shrinks in amplitude and merges with an equilibrium state. SN is a saddle-node bifurcation of equilibrium states, in which two equilibria (rest states) emerge. SNIC is a saddle-node bifurcation of equilibria on invariant circle, which is the same SN bifurcation that occurs on a limit cycle and, therefore, interrupts it. (B) F-I curves for three values of gh: gh = 0mS/cm^2, gh = 3.5mS/cm^2 and gh = 7mS/cm^2. (C1-3) Representative one-parameter bifurcation diagrams of three different bifurcation scenarios occurring at gh = 0mS/cm^2, 0<gh<7mS/cm^2 and gh> = 7mS/cm^2. Black curves represent equilibria. Red curves represent minima and maxima along a limit cycle. Solid stands for stable and dashed for unstable solutions. Dotted arrows represent bifurcation transitions. In C1, spiking is blocked as Iapp decreases through the LC Fold bifurcation. If Iapp increases through the same interval, the LC Fold bifurcation stays unnoticed because the equilibrium state remains stable, and only after the A-H bifurcation, spiking emerges. This is called hysteresis, and it creates bistability, in which either spiking or the rest state can be observed depending on the initial conditions for the voltage and Ca^2+^ concentration. In C2, the curve of equilibrium states folds and two saddle-node bifurcations of equilibria occur. The equilibria interrupt the unstable limit cycle (homoclinic bifurcation), but do not affect the stable limit cycle yet. In C3, the equilibria interrupt the stable limit cycle and destroy it in the SNIC bifurcation.

DA neurons are often identified by a prominent hyperpolarization-activated cation current (Ih), which gives a voltage “sag” upon injection of a hyperpolarizing current. It has been shown that Ih-expressing DA neurons exhibit smooth frequency decrease, pointing to a type I excitability [35,36]. However, the excitability type has been shown to vary depending on the intrinsic currents and network connectivity [42,43]. For example, modeling results suggest that changes in the intrinsic currents, e.g. L-type Ca^2+^ current, can switch the excitability type of the DA neuron [44,45]. Our model suggests that DA neurons display type II excitability in the presence of the Ih current. However, at small values of Ih conductance (gh = 1–6 mS/cm^2), the model behaves very similarly to the one without the Ih current, which might be taken for type I excitability in experiments. For small values of Ih conductance, the firing frequency of the simulated DA neuron decreases abruptly with the increase in the hyperpolarizing current. However, the discontinuity occurs at very low frequencies (see Fig 7), which might appear still as a gradual frequency decrease in the experiments. As the strength of Ih conductance increases (gh>6 mS/cm^2) the discontinuity in the F-I curve becomes clearer. For high conductances of Ih, low frequency tonic firing cannot be produced, which might affect the basal DA levels. This apparent switch in the excitability type might be important in the altered states of the DA system, when the Ih current is potentiated, for example, by EtOH [53,54].

As we describe earlier, the smooth frequency decrease and the transition to the rest state upon application of hyperpolarizing current or activation of GABAR occurs via a SNIC bifurcation (Fig 7C3). As soon as we include the Ih current into the model, the SNIC bifurcation splits into a saddle-node bifurcation of limit cycles (LC Fold) and two subcritical Andronov-Hopf bifurcations as shown in a two-parameter bifurcation diagram in fig 7A (see caption for the definitions of bifurcations). Thus, the excitability type changes to type II as we introduce Ih. However, for small values of its conductance gh, the two bifurcations are very close together and nearly indistinguishable, thus, the discontinuity in the F-I curve is in a very narrow range close to zero Hz, producing an illusion of a smooth transition to the rest state.

The bifurcation scenario for the values of gh less than 7mS/cm^2 is complex, although this complexity does not affect experimental observations as the affected limit cycle is unstable. As the magnitude of the negative applied current increases, the unstable limit cycle emerging from the Andronov-Hopf bifurcation, disappears as it collides with a saddle equilibrium state in a homoclinic bifurcation (not marked) and then appears again as the saddle disappears (SN1 in fig 7). The amplitude of the unstable limit cycle grows with the further increase in the negative applied current and, finally, it merges with the stable limit cycle for spiking and annihilates it at the fold bifurcation for limit cycles (LC Fold, Fig 7C2). For higher values of gh>7mS/cm^2 the curve of equilibrium states becomes monotonic, the saddle-node bifurcations of equilibrium states disappear and the transition from spiking to the rest state occurs via the LC fold bifurcation (Fig 7C1). Fig 7 illustrates representative examples of one-parameter bifurcation diagrams and F-I curves for all three cases: gh = 0, 0<gh<7 and gh> = 7.

For a range of negative applied currents, a stable limit cycle coexists with a stable equilibrium state, creating bistability. Thus, depending on the initial conditions, the neuron will be either in a rest (at equilibrium point) or in a repetitive spiking state (at limit cycle). The bistability region grows with the increase in the conductance of the Ih current (Fig 7A). Variations in current strength back and forth across this range will cause the neuron to jump from one state to the other. To check predictions generated by our model regarding the presence of hysteresis with respect to the applied current, slowly rising and falling current ramps can be applied to the DA neuron to switch it from the rest state to the repetitive firing mode and vice versa. The switch points should be different for falling and rising stimuli as shown in fig 7B. Despite the range of current intensities for coexistence is small relative to the total range for repetitive firing, it affects neuron behavior near the threshold. For example, once the current intensity reaches the value required for the onset of repetitive spiking, small perturbations of current will not switch the neuron back to the rest state. Thus, the presence of hysteresis makes spiking near the threshold more robust.

### Changes in the type of excitability caused by synaptic inputs

*In vivo*, the type of excitability may change due to tonic synaptic inputs [42], and next we explore how this change occurs in the DA neurons. AMPA receptor may co-activate together with NMDA and GABA receptors *in vivo*. By contrast to NMDAR, conductance of which is voltage-dependent, AMPA and GABA receptor currents are purely ohmic. Their combination is also an ohmic current:

$$I_{AMPA}+I_{GABA}=I_{eff}=g_{eff}(E_{eff}-v),$$

**Fig 8:**
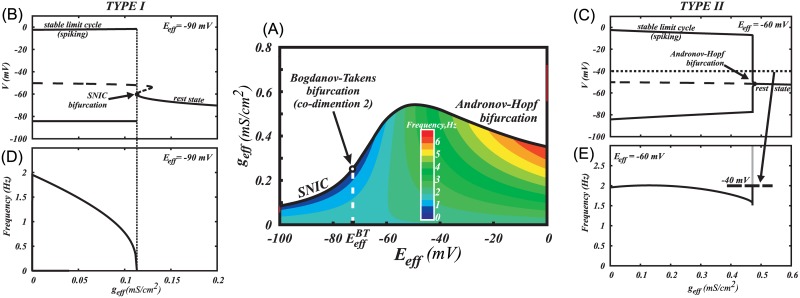
A combinations of ohmic synaptic conductances determines the type of excitability for the neuron. (A) The type of excitability is connected with the type of bifurcation at the transition from spiking to the rest state. The bifurcation type changes at $E_{eff}^{BT}=-72.26mV$, which is called Bogdanov-Tackens point. B) SNIC bifurcation scenario for a low reversal potential of the ohmic synaptic current (*E*_*eff*_ = −90*mV*), (D) A smooth frequency decrease to 0 upon application of combined synaptic conductance, corresponding to type I excitability. (C) An Andronov-Hopf bifurcation scenario for the higher reversal potential of the ohmic current (*E*_*eff*_ = −60*mV*), (E) An abrupt frequency decrease to 0, corresponding to type II excitability.

Where *g*_*eff*_ = *g*_*AMPA*_ + *g*_*GABA*_ is a combined synaptic conductance, and

$$E_{eff}={\frac{g_{GABA}E_{GABA}+g_{AMPA}E_{AMPA}}{g_{GABA}+g_{AMPA}}|}_{E_{AMPA}=0}=\frac{g_{GABA}}{g_{GABA}+g_{AMPA}}E_{GABA}$$

is a synaptic reversal potential. Fig 8 shows the frequency distribution and the type of bifurcation at the transition to the rest state on the plane of these two parameters: conductance and the reversal potential of the ohmic synaptic current. For instance, if the AMPA receptor is blocked, the reversal potential coincides with the GABAR reversal potential, which is in the range of -90 mV to -70 mV [55]. In this range, an increase in the conductance *E*_*eff*_ leads to a gradual decrease in the frequency to zero and a transition to the rest state via a SNIC bifurcation. By contrast, at higher reversal potentials, the frequency drops to zero abruptly and the transition corresponds to an Andronov-Hopf bifurcation. This suggests a transition to type II excitability for the DA neuron. Thus, elevated GABAR reversal potential or tonic activation of AMPAR leads to a switch in the excitability to type II.

### Activation of GABAR cannot rescue firing blocked by tonic AMPA

**Fig 9:**
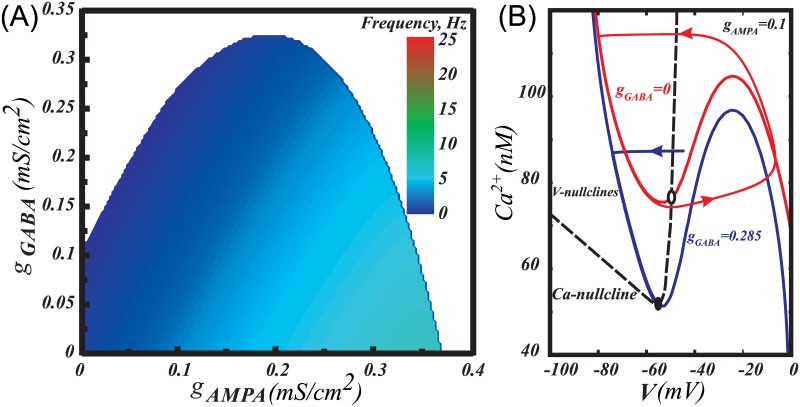
GABA cannot rescue firing blocked by tonic AMPA when GABA and AMPA receptors are co-activated. (A) Firing persists in a much smaller range of GABAR conductance when GABAR is co-activated with AMPAR as opposed to co-activation with NMDAR. (B) The analysis shows that AMPAR conductance transforms the voltage nullcline (solid) differently from NMDAR conductance. This transformation cannot be compensated by GABAR activation, because GABAR activation shifts the voltage nullcline in the same direction as AMPAR activation, leading to an interruption of oscillations by a stable state.

AMPAR-mediated input induces depolarization block in the DA neurons and firing cannot be restored. This is consistent with the previous modeling studies showing that NMDA can elicit bursting [56–58] or burst envelope [59], while AMPA abolishes high frequency firing. The dynamical explanation is that AMPAR activation shifts the minimum of the voltage nullcline across the Ca^2+^ nullcline, so that for high AMPAR conductance values (as well as positive applied currents), voltage oscillations decrease in amplitude and depolarization block occurs (S3B Fig). Thus, DA neuron firing does not exceed the frequency of ~10 Hz when driven with AMPAR activation, similarly to the experimental results (see e.g. [37,38,40]), Application of GABAR-mediated input shifts the voltage nullcline even further to the right and makes the stable equilibrium more robust. Therefore, the region of parameters for which spiking is produced is much smaller for combined application of AMPA and GABA than for combined NMDA and GABA activation (compare Figs 2B and 9A). Therefore, the prediction of our model is that a disinhibition burst can be supported by tonic background activation of NMDA but not AMPA receptors. Please note that nullclines can be produced only for the model without the fast sodium current (see S3 Fig for the illustration of depolarization block in the model with spike-producing currents).

### Activation of NMDAR shifts the boundary between the excitability types to lower values of the GABA reversal potential

**Fig 10:**
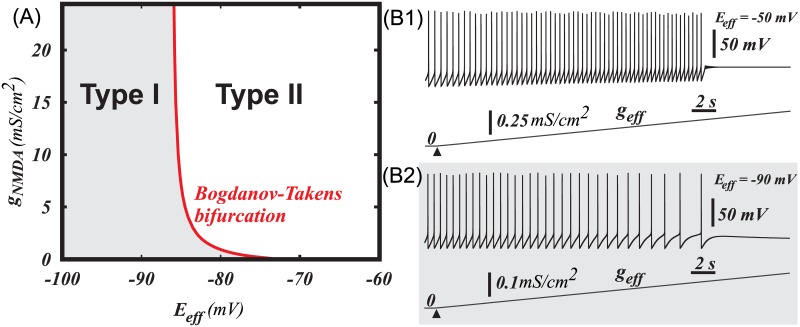
Tonic activation of NMDAR may change the excitability type from the first to the second. (A) The boundary between type I and type II excitability in the DA neuron shifts down with respect to the reversal potential of an ohmic synaptic current as NMDAR conductance grows. (B1-2) Two voltage traces illustrating the excitability types. For any synaptic reversal potential *E*_*eff*_ and NMDAR conductance from the grey region of type I excitability on panel (A), ramping the ohmic synaptic conductance *g*_*eff*_ increases the interspike intervals and then blocks firing. For the parameters from type II region, ramping the ohmic synaptic conductance blocks firing without the decrease in the frequency.

Together with AMPA and GABA receptors, the NMDAR may be also co-activated, since glutamate binds to both AMPA and NMDA receptors. To make the analysis of the excitability type possible in the parameter space of all three synaptic currents, we further perform 2-dimensional bifurcation analysis. The point marked Bogdanov-Takens bifurcation in Fig 8 is a good predictor of the type of excitability at the boundary between spiking and the rest state. Mathematically, it is defined as a junction of the SNIC bifurcation and the Andronov-Hopf bifurcation, as it appears in the figure. In Fig 10, we plot this point as a function of the NMDA receptor conductance. As in the previous figure, the transition to quiescence occurs as the combined conductance of the ohmic synapses *g*_*eff*_ grows. The information about the specific value of the conductance is omitted in Fig 10 because the transition occurs in a dimension orthogonal to the plane of the figure. For example, the transition in Fig 8 is represented by one line at *g*_*NMDA*_ *= 0*. The diagram in Fig 10 shows that the separation between the types of excitability shifts to lower values of the combined reversal potential for the AMPAR and GABAR currents as the NMDAR conductance increases. However, this shift quickly saturates and is restricted to the range of GABAR reversal potentials. Thus, similar to Ih, NMDAR activation may switch the type of excitability of the DA neuron from type I to type II in a certain window of other synaptic currents received by the neuron.

### DA release and synchronization in a population of heterogeneous DA neurons is influenced by tonic background synaptic currents

**Fig 11:**
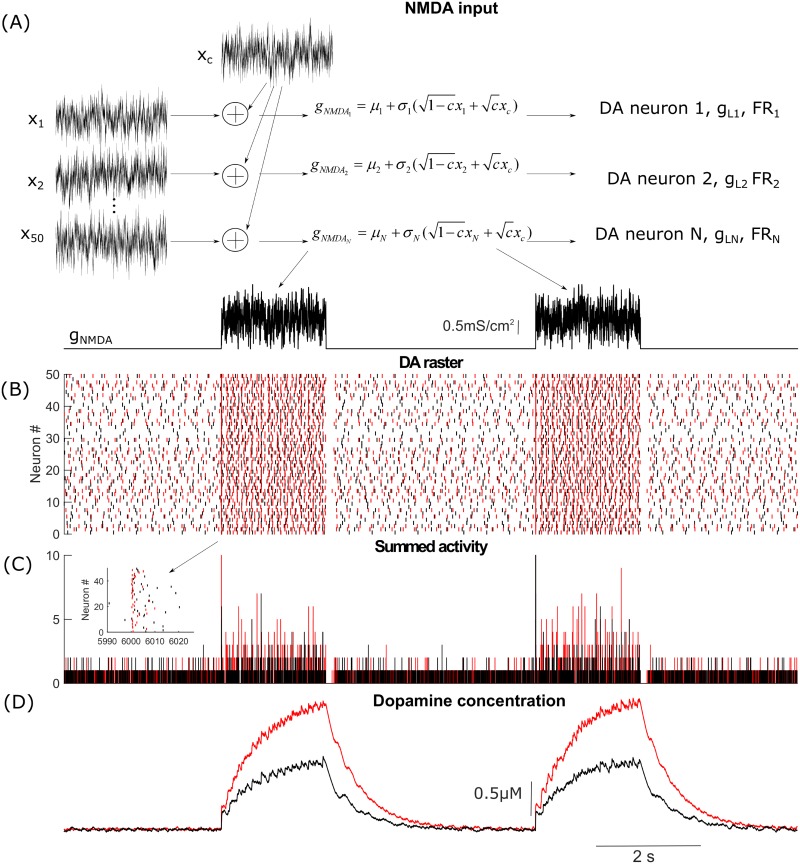
DA release and robustness of synchrony in a population of heterogeneous DA neurons are sensitive to background synaptic tone. (A) Stimulation paradigm in which DA neurons receive correlated fluctuating NMDA synaptic inputs with means of NMDAR conductances *μ*_1_ = *μ*_2_ = … = *μ*_*N*_ = 1.5*mS*/*cm*^2^ and standart deviations *σ*_1_ = *σ*_2_ = … = *σ*_*N*_ = 0.5*mS*/*cm*^2^. Fluctuations were modeled by Ornstein-Uhlenbeck process with *τ* = 5*ms* [33,64]. (B) Raster plot showing the firing of two heterogeneous populations of DA neurons in response to the fluctiating NMDA input. DA neurons shown in black do not receive tonic synaptic inputs, while neurons shown in red receive tonic AMPA and GABA inputs. The input are balanced such that the firing frequency remains the same. (C) Summed activity of the DA neurons in both populations. The level of synchrony during application of NMDA bursts is higher in the population receiving the tonic synaptic inputs. This is also illustrated in the insert zoomed in on the neural activity at the beginning of the burst. (D) Cumulative synaptic DA concentration produced by activity of the DA population with (red) and without (black) tonic background synaptic inputs. The DA neuron population that recieves tonic inputs releases more dopamine in response to NMDAR stimulation.

To illustrate the importance of changing DA neuron type of excitability, we simulated heterogeneous populations of DA neurons under two conditions: 1) in control (in the absence of the tonic synaptic inputs), and 2) during the tonic influence of AMPA and GABA inputs. The DA population is electrophysiologically heterogeneous [60–62], and its uncoordinated activity produces a homogeneous low-level DA concentration. In order to have similar firing rates and basal DA levels in both cases, we balanced the increase in firing rate produced by the application of AMPAR conductance with GABAR conductance (note that GABA can balance AMPA only for a very limited range of values). In both cases, DA neurons received correlated fluctuating NMDA inputs (Fig 11A, see methods for the detailed description of the inputs). Our simulations show that the population of DA neurons that receive the background synaptic tone produces higher DA levels in response to bursty correlated NMDA input than a population without the synaptic tone (Fig 11D). As described above, DA neurons are type I excitable in the absence of synaptic tone, while AMPAR activation switches DA neuron excitability to type II. Thus, the transition from type I to type II excitability of the DA neurons is accompanied by higher dopamine release in response to a correlated synaptic input. The higher responsiveness is partially due to a greater synchronization of the DA neurons receiving the synaptic tone, as evident by the higher number of peaks in their summed activity in Fig 11C. Type II neurons display more robust synchrony when they receive a common input, even in the presence of independent noise [33,63]. Thus, *in vivo* background synaptic tone might be important not only for regulating basal DA levels, but also for the responsiveness of the DA neurons, so that they are more ready to produce coincident bursts in response to correlated synaptic inputs.

## Discussion

### The excitability type of the DA neuron

The type of excitability for a neuron determines the neurons’ responses to stimuli and their dynamics in a population (phase response curve, synchronization, resonators vs. integrators). The type of bifurcation determines the type of excitability: neural oscillations that arise via an Andronov-Hopf bifurcation have type II excitability, while those appearing via SNIC have type I excitability [52,65]. Based on the bifurcation analysis and frequency responses to hyperpolarizing inputs (negative injected current and GABAR conductance), we have shown that in control conditions, the simulated DA neuron is type I-excitable. It’s known that the type of excitability *in vivo* may be different from *in vitro* [42]. *In vivo*, DA neurons display irregular low-frequency activity occasionally interrupted by high-frequency bursts. This low-frequency regime may reflect the balance of inhibitory and excitatory inputs. We found that, in the most prominent low-frequency regime, the DA neuron is type I excitable, in either high or low synaptic conductance states.

The baseline level of dopamine is important for the normal function of the brain. The level is determined by the background activity of the DA neurons. This activity is intrinsically generated by the neuron and controlled by its synaptic inputs [66], reviewed in [67]. Thus, the capacity of the DA neuron to adjust its firing rate according to the inputs is vital. The graded response curve of a type I-excitable neuron, as opposed to an on-off response of a type II neuron, provides this capacity. Accordingly, at every level of excitation provided by NMDAR input, inhibitory GABA synaptic conductance can balance it and bring the frequency down to an arbitrary low value. A similar hyperpolarizing current activated by dopamine D2 receptors on the DA neuron has also been shown to slow down its firing rather than abruptly block it all together [68]. These are very important autoregulatory functions of the DA system that allow it to adjust basal DA levels in target areas.

### The role of the subthreshold sodium current

Persistent sodium current is known to amplify subthreshold oscillations [69] and increases neural excitability of DA neurons by contributing to spontaneous depolarization in between the spikes [25]. Consistent with experimental observations, the subthreshold sodium current increases the firing rate of the DA neuron in the model. Additionally, we found that the current is necessary for achieving gradual frequency decrease upon application of hyperpolarizing current, thus, maintaining type I excitability of the DA neuron. The type of excitability is determined by the internal properties of the currents contributing to pacemaking in the DA neuron. L-type Ca^2+^ and SK-type Ca^2+^-dependent K^+^ currents are the core currents that traditionally constitute this mechanism [7–18] (but see the section on the mechanisms of DA neuron pacemaking). However, our model shows that the mechanism results in type II excitability, in which a hyperpolarizing current blocks the voltage oscillations without restoring a low frequency. Our explanation is that, without the contribution of further currents, the steep part of the Ca^2+^ nullcline is very close to the minimum of the voltage nullcline (Fig 4A), because they reflect the same event: opening of the Ca^2+^ channel. This positions the system close to the Andronov-Hopf bifurcation, which occurs whenever the minimum of the voltage nullcline moves across the Ca^2+^ nullcline. When the subthreshold sodium current is included into the mechanism, the minimum of the voltage nullcline reflects opening of this current, and it is shifted away from the steep part of the Ca^2+^ nullcline (Fig 6B). This shifts the system away from the Andronov-Hopf bifurcation. Now, a downward shift of the voltage nullcline following inhibitory inputs moves its minimum across the flat part of the Ca^2+^ nullcline and produces a SNIC bifurcation. In this transition, the firing frequency gradually reduces to zero, and this allows a balance between NMDAR and GABAR conductances and restores the background firing frequency.

### Related studies of NMDA-GABA balance in DA neurons

The influence of NDMA and GABA receptor conductances on the DA neuron have been studied in several papers [23,22,70]. The compensatory influence of NDMA and GABA receptor activation on the firing frequency has been predicted in modeling studies by Komendantov et al. [23]. Lobb et al. [70] modified our previous model [24] to capture the balance of the inhibition and excitation and disinhibition bursts. In these models, a high maximal frequency (>10 Hz) can be achieved by tonic activation of the AMPAR or in response to depolarizing current injection, which contradicts experimental observations [7,34]. To the contrary, a number of experimental studies suggest that stimulation of NMDA receptors evokes a burst of high-frequency firing, whereas AMPA receptor activation evokes modest increases in firing [37–40] (but see [61,71]). This is an important distinction, which impacts the excitability of the neuron. Further, in Komendantov et al. [23] and Canavier & Landry [22], the NMDAR conductances were restricted to dendrites, whereas GABAR conductance was somatic. The mechanism of frequency rise during dendritic application of NMDA is different from the mechanism of response to somatic NMDAR stimulation [56,24]. Somatic NMDAR stimulation has been shown to elicit high-frequency firing in earlier experiments [46,40] and used to achieve the NMDA-GABA balance [35]. Here, we base a new model on our previous model [56] that presented a mechanism for somatically-induced high-frequency firing in a reconstructed morphology first and reduced it to a single compartment. In the current model, we have integrated the mechanism for high-frequency firing together with the balance of NMDAR and GABAR activation.

### Mechanisms of DA neuron pacemaking

The mechanism of low frequency pacemaking in the DA neurons has been extensively studied. However, it is still a matter of on-going debate in the literature since different experimental results lead to contradicting conclusions, proposing that different currents are critical for the DA neuron spontaneous firing. In a number of experimental and modeling studies it has been shown that spontaneous tonic firing relies on the interactions between the voltage gated calcium (Ca^2+^) and SK-type Ca^2+^ -dependent potassium (K^+^) currents [7–19]. Wilson and Callaway [4] and later Chan et al. [19] showed that calcium-driven slow oscillatory potentials (SOPs) drive the spiking rate of the SNc DA neurons. Chan et al 2007 [19] also showed that dependence of pacemaking on Ca^2+^ oscillations changes with the age. Particularly, TTX blocks slow oscillations in juvenile neurons, but not in adult neurons, which is related to the change in density of Ca^2+^ channels with age. Ping and Shepard 1996 showed that the frequency of SOPs after the application of TTX is approximately the same as the frequency of spiking. In contrast, in a more recent study, Guzman et al. [26] demonstrated that, in a number of DA neurons, SOPs and spiking frequencies are weakly correlated, and that TTX inhibits spontaneous oscillatory potentials pointing to the importance of sodium currents for pacemaking. A number of other studies also suggest that sodium channels are highly involved in controlling spontaneous DA neuron frequency [26,25], especially in the VTA DA neurons [27].

The sources of the apparent discrepancy in the experimental results were investigated by Drion and colleagues [21]. Based on the combination of experimental and modeling approaches, authors suggested that calcium and sodium currents likely cooperate to produce pacemaking and prevailing mechanism depends on the density of the ion channels in the neuron. Further, authors showed that the lack of correlation between spikes and SOPs does not lead to a conclusion that generating mechanisms are different. The complementary role of the two mechanisms, notably if they co-exist in the same cell or represent pacemaking in distinct populations, is a matter of on-going research in the field.

For the sodium-based pacemaking mechanism, it is still unclear what hyperpolarizing current provides the long interspike interval when the SK current is not functional. This renders the Ca^2+^-independent oscillatory mechanism incomplete and is a matter of a future investigation. Control of the firing by the SK current has been shown to be stronger in the DA neurons positioned more laterally in the midbrain [15]. Thus, we focus on the subpopulation of DA neurons that are more abundant in the substantia nigra pars compacta (SNc) than the ventral tegmental area (VTA).

### Intrinsic Ih current and synaptic inputs can switch the excitability type of the DA neuron

Changes in intrinsic currents can affect the excitability type and, thus, computational properties of the DA neuron. For instance, we observed that potentiation of Ih current promotes type II excitability of the simulated DA neuron (Fig 7). Further, we show that Ih current can induce bistability in the DA neuron, and the bistability region increases with the increase in Ih conductance (Fig 7B). This can affect the behavior of the neuron near the boundary between spiking/resting states, particularly, if the current reached the value necessary to induce spiking onset, a small perturbation in the current will not silence the neuron. This makes spiking more robust near the threshold. In addition to the contribution of Ih current to pacemaker activity, has been shown in DA neurons [72], as well as in the other neuronal types that Ih induces intrinsic subthreshold resonance [73–75]. Thus, augmentation of Ih current increases oscillatory behavior of the DA neurons, as well as their synchronization in response to excitatory pulses. However, low-frequency tonic firing could not be maintained at high conductances of Ih current, likely affecting background DA levels.

Further, the influence of tonic synaptic inputs can also change the transition to the rest state and, therefore, be described as altered excitability. Tonic activation of AMPA receptors or an elevated reversal potential of the GABAR conductance may make the low-frequency balanced state unreachable. The reason for that is a transition to type II excitability: firing is blocked at higher frequencies. A model prediction that follows from this result is that tonic AMPAR activation induces depolarization block and firing cannot be rescued by application of GABA. Our explanation is that shunting is so strong that opening of the subthreshold sodium current cannot sustain the voltage growth. In other words, these changes unfold the voltage nullcline and bring its minimum close to the steep part of the Ca^2+^ nullcline (Fig 9B). This primes the system for the Andronov-Hopf bifurcation responsible for type II excitability. Further, we found that NMDAR activation also biases the neuron towards type II excitability (Fig 10). Although the type may change as parameters shift away from the boundaries of the firing region [76], together, these results suggest that in high-frequency regimes the DA neuron displays type II excitability. This switch in excitability type may play a role in a transient increase in DA concentration in response to salient stimuli as it is easier to synchronize type II neurons by an excitatory input. Fig 11 supports this hypothesis by showing higher transient DA release produced by heterogeneous population of DA neurons receiving synaptic AMPA and GABA tones than in the absence of synaptic tone. Thus, correlated excitatory synaptic inputs are more likely to evoke robust coincidence DA release when DA neurons display type II excitability.

### Implications of the DA neuron excitability type on computations and possibly different behavioral states

A growing body of literature links the type of excitability to neural coding [29,31,41,65,77–79]. For instance, Prescott et al. [80] suggested that type I neurons are best suited for coding stimulus intensity. Hence, the DA neuron is designed for encoding the intensity of the tonic depolarizing and hyperpolarizing inputs by its smooth frequency dependence. This further supports and augments a recently found unique computational role for the DA neuron: it performs subtraction of inhibitory and excitatory inputs [81]. The operation is optimal to calculate unpredicted value of an event but rarely observed and hard to implement in the brain. The first type of DA neuron excitability is necessary to quantitatively encode the level of input by the firing rate and perform the subtraction.

**Fig 12:**
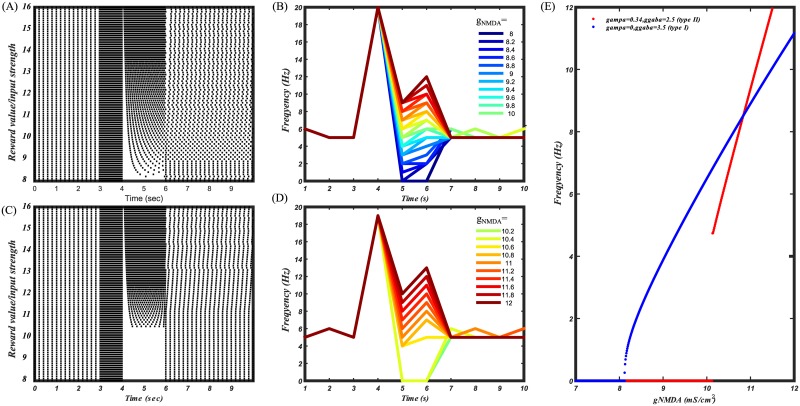
Coding of the reward value by type I and type II DA neurons. (A) Raster of type I DA neuron in response to gradually increasing input strengths/ reward value. (B) Frequency responses of the type I DA neuron to different reward values. (C) Raster of the type II DA neuron in response to gradually increasing input strengths/ reward values. (D) Frequency responses of type II DA neuron to different reward values. (E) F-I curves of type I (blue) and type II (red) DA neurons. Due to a discontinuity in the F-I curve, type II DA neurons are unable to encode low reward values.

Several studies, for example Eshel et al. 2016 [82] and Tobler et al. 2005 [83], showed that activation of DA neurons gradually increases with the increase in the reward value. We attempted to reproduce this experimental result in our model. For the simulations, we assumed that the reward value is proportional to the strength of NMDA input coming to the DA neurons (*g*_*NMDA*_). GABA inputs do not seem to be a plausible candidate because Eshel et al. 2016 [81] showed that firing of the GABA neurons, as opposed to the DA neurons, does not vary consistently with the reward value. We calculated firing frequency dependence of the type I/ type II DA neurons on the input strength (Fig 12). Type II DA neurons were modeled by applying tonic AMPA along with NMDA conductance (additional excitation was compensated by increasing GABA conductance). Type II DA neurons are unable to encode low reward values as their firing abruptly drops to zero with the decrease in the input strength. In contrast, frequency dependence of type I DA neurons resembles the experimental curve of the DA neuron frequency dependence on the reward value shown in [82]. The difference can also be seen in the raster (Fig 12A and 12C) and frequency responses (Fig 12B and 12D) to a transient increase in the input strength, representing the reward value. First peak in the frequency response represents salience, and was simulated by applying constant level of NMDA for all the values, while second peak represents the reward value, and was simulated by scaling NMDA input accordingly. There is a gap in a frequency response to low reward values of type II DA neurons (Fig 12D). Thus, type I DA neurons best encode the value of an event, i.e. the difference between predicted and received reward.

On the other hand, spike timing of type I neurons in response to weak or noisy transient inputs is not reliable [84,85], while temporal precision of type II neurons is much higher. The ability of the DA neuron to switch excitability type from type I to type II under certain synaptic inputs might play a significant role in producing enhanced transient DA release, since it likely relies on the precise coordinated activity of the DA neurons. Multiple drugs of abuse, including EtOH (e.g. [86]) evoke transient increases in the DA concentration in nucleus accumbens.

**Fig 13:**
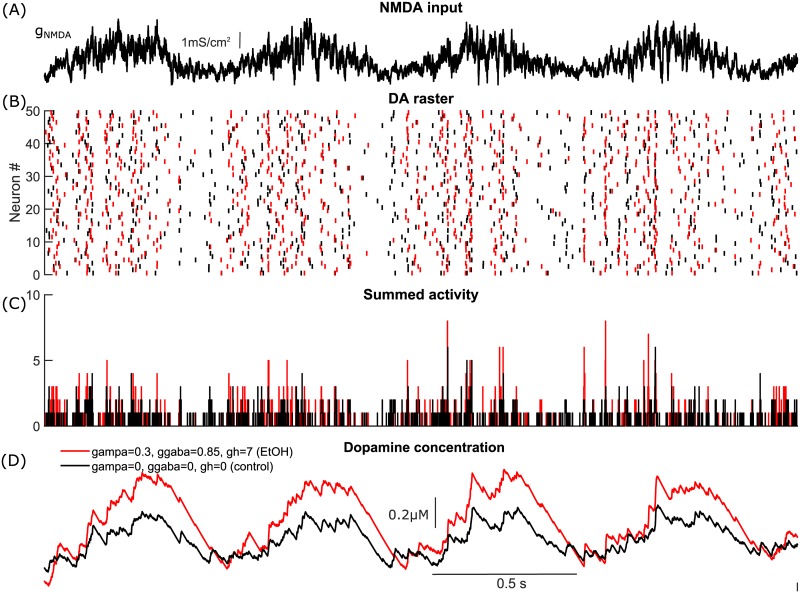
EtOH switches DA neurons to type II and induces higher DA transients. A) DA neurons receive correlated fluctuating NMDA synaptic inputs modulated by a sine wave of 1 Hz. The fluctuations were modeled by Ornstein-Uhlenbeck process with *τ* = 5*ms*. (B) Raster plot showing the firing of two heterogeneous poulations of DA neurons in response to the fluctiating NMDA input. DA neurons shown in black are type I, neurons shown in red are type II (under EtOH). (C) Summed activity of the DA neurons in both populations. The higher peaks signify that the level of synchrony in the population of type II neurons is higher. (D) Cumulative synaptic DA concentration produced by the activity of the type II DA neurons (red) and type I DA neurons (black). The DA neuron population modeled under EtOH conditions releases more dopamine in response to NMDAR stimulation.

Using our model, we show that EtOH shifts excitability of the DA neurons to type II and induces higher DA release. EtOH acts on multiple intrinsic and synaptic currents. Mainly, it enhances I_h_ current [87,88] and increases AMPA/NMDA ratio [89]. Moreover, it increases GABA release onto DA neurons [90]. Thus, we modeled EtOH action by increasing I_h_, AMPA and GABA receptor currents. Ih and AMPAR currents switch DA neuron excitability to type II and, therefore, promote synchronization in the population of DA neurons in response to noisy excitatory inputs (Fig 13). This is one of the mechanisms that can produce higher DA transients. By switching DA neuron to type II, EtOH can increase the motivational potential of a stimulus because the same excitatory input produces enhanced DA signal under EtOH. In other words, neutral stimulus can become salient after EtOH exposure. Our modeling prediction regarding the excitability switch after EtOH could be tested *in-vitro* by studying how the shape of F-I curve changes after EtOH. In *in-vivo* conditions it could be checked by stimulating DA neurons before and after EtOH exposure with a chirp pattern signal in order to check for spiking resonance. To test whether DA neurons better synchronize after EtOH, a phase response curve (PRC) or a spike triggered average (STA) could be calculated. Presence of the negative PRC component and narrow STA indicates that neurons are more amenable to synchronization by common synaptic noise.

In conclusion, our results predict that DA neurons can exhibit traits of both integrators and resonators and these traits are modulated by intrinsic and synaptic conductances. Depending on the current constitution, DA neurons can perform rate coding by integrating slow variations in the inputs and adjust basal DA concentration or they can detect transient coherent changes in the inputs and synchronize for producing robust DA transients.

## Methods

The biophysical model of the DA neuron is a conductance-based one-compartmental model modified from [91]

$$\begin{array}{l} c_{m}\frac{dv}{dt}=\overset{I_{Ca}}{\overset{︷}{g_{Ca}(v)(E_{Ca}-v)}}+\overset{I_{KCa}+I_{K}+I_{DR}}{\overset{︷}{\left(g_{KCa}([Ca^{2+}])+g_{K}(v)+{\bar{g}}_{DR}n^{4})(E_{K}-v\right)}}+\\ +\underset{I_{sNa}+I_{Na}}{\underset{︸}{\left(g_{sNa}(v)+{\bar{g}}_{Na}m^{3}h)(E_{Na}-v\right)}}+\underset{I_{leak}}{\underset{︸}{g_{l}(E_{l}-v)}}+\underset{I_{h}}{\underset{︸}{g_{h}q(E_{h}-v)}}+\underset{I_{syn}}{\underset{︸}{I_{NMDA}+I_{AMPA}+I_{GABA}}},\\ \frac{d[Ca^{2+}]}{dt}=\frac{2\beta }{r}\left(\frac{\left(g_{Ca}(v)+0.1g_{L}\right)}{zF}(E_{Ca}-v)-P_{Ca}[Ca^{2+}]\right),\\ \frac{dq}{dt}=\frac{q_{\infty }-q}{\tau_{q}(v)},\\ \frac{dh}{dt}=\alpha_{h}(v)(1-h)-\beta_{h}(v)h,\\ \frac{dn}{dt}=\alpha_{n}(v)(1-n)-\beta_{n}(v)n, \end{array}$$ (1)

where *v* is the voltage and *c*_*m*_ is the membrane capacitance. There are eight intrinsic currents of the DA neuron: a calcium current (*I*_*Ca*_), a calcium-dependent potassium current (*I*_*KCa*_), a potassium current (*I*_*K*_), a direct rectifier current (*I*_*DR*_), a subthreshold sodium current (*I*_*sNa*_), a hyperpolarization-activated current (*I*_*h*_), a fast sodium current (*I*_*Na*_), and a leak current (*I*_*leak*_). The first subgroup of intrinsic currents: *I*_*Ca*_, *I*_*KCa*_, *I*_*K*_, *IsNa* and *I*_*h*_ constitute a pacemaking mechanism of the DA neuron. The second subgroup of the intrinsic currents (*I*_*Na*_, *I*_*DR*_) is responsible for spike generation. The last subgroup includes synaptic currents: the excitatory α-Amino-3-hydroxy-5-methyl-4-isoxazolepropionic (AMPA) and *N*-Methyl-D-aspartate (NMDA) receptor currents (*I*_*AMPA*_ and, *I*_*NMDA*_ respectively) and the inhibitory γ-Aminobutyric acid (GABA) receptor current (*I*_*GABA*_). Synaptic inputs can produce bursts and pauses in firing.

### Intrinsic oscillator

The main currents of the model that produce pacemaking activity of DA neuron are an L-type voltage-dependent calcium current (*I*_*Ca*_) and an SK-type calcium-dependent potassium current (*I*_*KCa*_). Gating of the calcium current is instantaneous and described by the function:

$$g_{Ca}=\bar{g_{Ca}}\cdot \frac{\alpha_{c}^{4}(v)}{\alpha_{c}^{4}(v)+\beta_{c}^{4}(v)}$$ (2)

Calibration of the calcium gating function reflects an activation threshold of an L-type current, which is significantly lower in DA cells than in other neurons (~ -50mV; [4]). Calcium enters the cell predominantly via the L-type calcium channel. Contribution due to the NMDA channel is minor [92]. Thus, calcium concentration varies according to the second equation of the system (1). It represents balance between Ca^2+^ entry via the L channel and a Ca^2+^ component of the leak current, and Ca^2+^ removal via a pump. In the calcium equation, *β* is the calcium buffering coefficient, i.e. the ratio of free to total calcium, r is the radius of the compartment, *z* is the valence of calcium, and *F* is Faraday’s constant. *P*_*ca*_ represents the maximum rate of calcium removal through the pump. A large influx of Ca^2+^ leads to activation of the SK current, which contributes to repolarization as well as afterhyperpolarization of the DA cell. Dependence of the SK current (*I*_*KCa*_) on calcium concentration is modeled as follows:

$$g_{K,Ca}=\bar{g_{K,Ca}}\cdot \frac{{\left[Ca^{2+}\right]}^{4}}{{\left[Ca^{2+}\right]}^{4}+{\left[K^{+}\right]}^{4}}$$ (3)

The neuron is repolarized by the activation of a large family of voltage-gated potassium channels. In addition to the already described potassium current, the model contains voltage-dependent K current (*I*_*K*_). Conductance of this current is given by a Boltzmann function:

$$g_{K}=\bar{g_{K}}\cdot \frac{1}{1+\text{exp}(\frac{-(v+10)}{7})}$$ (4)

The DA neuron expresses voltage-gated sodium channels that carry a large transient current during action potentials (a spike-producing sodium current) and a non-inactivating current present at subthreshold voltages (a subthreshold sodium current). Even though the persistent subthreshold sodium current is much smaller than the transient spike-producing current, it influences the firing pattern and the frequency of the DA neuron by contributing to depolarization below the spike threshold [25]. We modeled the voltage dependence of the subthreshold sodium current as follows:

$$g_{sNa}={\bar{g}}_{sNa}\frac{1}{1+\text{exp}(\frac{-(v+50)}{5})}$$ (5)

The kinetics and the voltage dependence of the subthreshold sodium current were taken from [93].

The majority of DA neurons express a hyperpolarization-activated nucleotide-gated (HCN) inward cation current (*I*_*h*_). The HCN current contributes to spontaneous firing of subpopulations of DA neurons [94]. The activation variable of *I*_*h*_ is governed by a first-order ordinary differential equation (the third equation of the system (1)).

The maximal activation of *I*_*h*_ current is described by the following voltage-dependent equation [88]

$$q_{\infty }=\frac{1}{1+\text{exp}(\frac{v+95}{8})}$$ (6)

The voltage-dependent time constant is described by

$$\tau_{q}=\frac{625\cdot \text{exp}(0.075(v+112))}{1+\text{exp}(0.083(v+112))}$$ (7)

The leak current (*I*_*leak*_) in the model has the reversal potential of -35 mV, which is higher than in the majority of neuron types. In DA neurons, several types of depolarizing, nonselective cation currents are expressed, which likely contribute to depolarization during interspike intervals.

### Model calibration

#### Subthreshold currents

Calibration of the subthreshold Ca^2+^-K^+^ oscillatory mechanism remained very similar to previously described [89]. Experiments show that subthreshold sodium currents contribute to depolarization towards the spike threshold [25,27]. Accordingly, the addition of the subthreshold sodium current into the model increases the background firing frequency of the DA neuron. However, in the model this leads to a significant increase in maximal frequencies achieved during application of a constant depolarizing current or AMPA, whereas in experiments these frequencies have been shown to be limited by approximately 10 Hz [34]. We preserve this limit in the model by accounting for a Ca^2+^ component of the leak current. Addition of the small Ca^2+^ leak current moves the flat part of the Ca^2+^ nullcline up and produces a slight upward bent of the Ca^2+^ nullcline, which was not essential for the SNIC bifurcation. This reinforced the negative feedback loop through the Ca^2+^-dependent K^+^ current and limited the maximal frequencies. NMDAR activation increases the frequency, and our calibration of the model allowed an inhibitory or hyperpolarizing input to reduce the frequency to an arbitrary low value. Ca^2+^ entry through NMDA receptor was omitted to implement the spatial segregation of NMDAR and SK channels as in the previous models [56,23].

#### Spike-producing currents

We included spike-producing sodium current with maximal conductance $g_{Na}(v)={\bar{g}}_{Na}m^{3}h$ and potassium delayed rectifier current with maximal conductance $g_{DR}=\bar{g_{DR}}n^{4}$. The activation of the Na^+^ current is assumed to be instantaneous and is described by the function

$$m(v)=\frac{\alpha_{m}}{\alpha_{m}+\beta_{m}},$$

$$where\;\alpha_{m}=\frac{-0.32(v+39)}{e^{-\frac{v+39}{4}}-1},\;\beta_{m}=\frac{0.28(v+4)}{e^{\frac{v+4}{5}}-1}.$$ (8)

The inactivation kinetics is described by the equation for *h* in System (1), where

$$\alpha_{h}=0.01e^{-\frac{v+47}{18}},\;\beta_{h}=\frac{1.25}{1+e^{-\frac{v+24}{5}}}$$ (9)

The delayed rectifier activation variable *n* is described by the 5th equation in System (1), where

$$\alpha_{n}=\frac{-0.0032(v+5)}{e^{-\frac{v+5}{10}}-1},\;\beta_{h}=0.05e^{-\frac{v+10}{16}}.$$ (10)

The currents are calibrated to produce a spike per each maximum of the voltage oscillations produced by the pacemaking mechanism without significantly changing the firing rate or pattern.

### Synaptic inputs

DA neurons receive glutamatergic (Glu) excitatory drive through AMPA and NMDA receptors and inhibitory drive through GABA receptors. Changes in the membrane potential induced by synaptic conductances are described by the following equation

$$I_{syn}=\overset{I_{NMDA}}{\overset{︷}{g_{NMDA}(v)sig(s_{NMDA}^{act})(E_{NMDA}-v)}}+\overset{I_{AMPA}}{\overset{︷}{g_{AMPA}sig(s_{AMPA}^{act}s_{AMPA}^{des})(E_{AMPA}-v)}}+\overset{I_{GABA}}{\overset{︷}{g_{GABA}s_{GABA}(E_{GABA}-v)}}$$ (11)

where *g*_*NMDA*_(*v*), *g*_*AMPA*_, *g*_*GABA*_ are the maximal conductances of NMDA, AMPA and GABA receptor currents accordingly, *s*_*NMDA*_, *s*_*AMPA*_, *s*_*GABA*_ are gating variables that depend on the input spike trains.

The AMPA and GABA conductances are voltage-independent, but the NMDA conductance has voltage sensitivity as in [95]

$$g_{NMDA}(v)=\frac{{\bar{g}}_{NMDA}}{1+0.1[Mg^{2+}]e^{-m_{e}v}}$$ (12)

where [*Mg*^2+^] denotes the amount of magnesium, taken to be 0.5μM. The low slope of the voltage dependence (*m*_*e*_ = 0.062) is critical for the increase in the frequency of spikes or subthreshold oscillations during NMDA application [56].

### Modeling of Glu and GABA asynchronous inputs to the DA neuron

#### Glu input

We started our study with investigating the influence of asynchronous synaptic inputs. Asynchronous Glu input to the DA neuron was produced by 35 Poisson distributed spike trains with frequencies of approximately 10 Hz. The number of spike trains was chosen to produce a relatively constant level of NMDA receptor activation, and, at the same time, take into account the effects of convergent synaptic inputs on the DA neuron, by thresholding NMDAR to activate only by coincidence of two or more spikes. The activation of the receptors in response to a synaptic input is described by the following equation

$$\frac{ds_{i}^{act}}{dt}=\frac{j(1-s_{i}^{act})}{\tau_{act}}-\frac{(1-j)s_{i}^{act}}{\tau_{deact}},$$ (13)

where *j* denotes a dimensionless synaptic input. It is normalized to change from 0 to 1 for 1 ms interval to mimic a single spike in the input. *i* denotes a receptor type, AMPA or NMDA.

#### GABA input

Asynchronous inhibitory input was produced by a population of 30 GABA neurons. The number of GABA neurons projecting to a DA neuron is not known. To choose this number, we note first that the percentage of GABAergic neurons varies between 12 and 45% in different sub regions of the ventral tegmental area (VTA) [96]. Since the GABA neurons powerfully modulate DA neuron activity via direct, monosynaptic inhibitory connections [55,97–100], one can expect multiple GABA neurons to make connections with a single DA neuron. Second, we found that our results are valid for a wide range of the number of GABA neurons and start to change only if the number becomes small (10 or lower) [91].

Voltage dynamics of each GABA neuron is described by the Wang-Buszaki equations of a fast spiking neuron [101],

$$c_{m}\frac{dv_{i}}{dt}={\bar{g}}_{Nag}m^{3}(v_{i})h(E_{Na}-v_{i})+{\bar{g}}_{Kg}n(E_{K}-v_{i})+g_{\text{lg}}(E_{\text{lg}}-v_{i}),$$ (14)

where *v*_*i*_ is a voltage of *i*_*th*_ GABA neuron in a population. The activation variable *m* is assumed fast and substituted by its steady-state function $m_{\infty }=\frac{\alpha_{m}}{\alpha_{m}+\beta_{m}}$, where $\alpha_{m}=\frac{0.1(v_{i}+30)}{1-\text{exp}(\frac{-(v_{i}+30)}{10})}$, $\beta_{m}=4\text{exp}(\frac{-(v_{i}+55)}{18})$. The inactivation variable *h* follows first-order kinetics, $\frac{dh}{dt}=\alpha_{h}(v)(1-h)-\beta_{h}(v)h,$ where $\alpha_{h}=0.07\text{exp}(\frac{-(v_{i}+53)}{20})$, $\beta_{h}=\frac{1}{1+\text{exp}(\frac{-(v_{i}+23)}{10})}$. The activation variable n obeys the following equation, $\frac{dn}{dt}=\alpha_{n}(v)(1-n)-\beta_{n}(v)n,$ where $\alpha_{n}=\frac{0.01(v_{i}+29)}{1-\text{exp}(\frac{-(v_{i}+29)}{10})}$ and $\beta_{n}=0.0875\text{exp}(\frac{-(v_{i}+39)}{80})$.

The parameters of these equations were calibrated according to experimental observations [34]. Intrinsic firing frequencies of these neurons were set to a range of 12–22 Hz, similar to what is observed experimentally [34,102,103]. The activity of each GABA neuron contributes to the activation of the GABAR current entering the DA neuron by increasing the gating variable according to the equation

$$\frac{ds_{GABA_{i}}}{dt}=\frac{g_{spike}(v_{i})(1-s_{GABA}{}_{i})}{\tau_{gact}}-\frac{(1-g_{spike}(v_{i}))s_{GABA_{i}}}{\tau_{gdeact}},$$ (15)

where $g_{spike}=\frac{1}{1+\text{exp}(\frac{-v_{i}}{2})}$

The total receptor activation is a sum of contributions produced by all GABA neurons in a population. A parameter that scales the GABA current is GABAR conductance *g*_*GABA*_. We normalize the GABA gating variable by the number of neurons in order to keep its value in a range between zero and one. Thus, for an asynchronous GABA population, the GABAR will be partially activated and the gating variable will have a low value. Model parameters are given in Table 1.

**Table 1:** Model parameters.

Parameter	Description	Value
*c*_*m*_	Membrane capacitance of Da and GABA neurons	1*μF*/*cm*^2^
${\bar{g}}_{K}$	Maximal potassium conductance on DA neuron	1*mS*/*cm*^2^
${\bar{g}}_{Ca}$	Maximal calcium conductance on DA neuron	2.5*mS*/*cm*^2^
${\bar{g}}_{KCa}$	Maximal calcium-dependent potassium conductance on DA neuron	7.8*mS*/*cm*^2^
${\bar{g}}_{sNa}$	Maximal subthreshold sodium conductance on DA neuron	0.13*mS*/*cm*^2^
*g*_*l*_	Leak conductance on DA neuron	0.18*mS*/*cm*^2^
${\bar{g}}_{Na}$	Maximal sodium conductance on DA neuron	50*mS*/*cm*^2^
${\bar{g}}_{DR}$	Maximal delayed rectifier conductance on DA neuron	2*mS*/*cm*^2^
${\bar{g}}_{Nag}$	Maximal sodium conductance on GABA neuron	22*mS*/*cm*^2^
${\bar{g}}_{Kg}$	Maximal potassium conductance on GABA neuron	7*mS*/*cm*^2^
${\bar{g}}_{NMDA}$	Maximal NMDA conductance on DA and GABA neurons	varied
*g*_*AMPA*_	AMPA conductance on DA and GABA neurons	varied
*g*_*GABA*_	GABA conductance on DA and GABA neurons	varied
*E*_*K*_	Potassium reversal potential on DA and GABA neurons	−90*mV*
*E*_*ca*_	Calcium reversal potential on DA neuron	50*mV*
*E*_*Na*_	Sodium reversal potential on DA and GABA neurons	55*mV*
*E*_*l*_	Leak reversal potential on DA neuron	−35*mV*
*E*_lg_	Leak reversal potential on GABA neuron	−51*mV*
*E*_*NMDA*_	NMDA reversal potential on DA and GABA neurons	0*mV*
*E*_*AMPA*_	AMPA reversal potential on DA and GABA neurons	0*mV*
*E*_*GABA*_	GABA reversal potential on DA neuron	−90*mV*
*τ*_*aact*_	AMPA receptor activation time on DA and GABA neurons	1*ms*
*τ*_*adeact*_	AMPA receptor deactivation time on DA and GABA neurons	1.6*ms*
*τ*_*nact*_	NMDA receptor activation time on DA and GABA neurons	7*ms*
*τ*_*ndeact*_	NMDA receptor deactivation time on DA and GABA neurons	170*ms*
*τ*_*gact*_	GABA receptor activation time on DA and GABA neurons	0.08*ms*
*τ*_*gdeact*_	GABA receptor deactivation time on DA and GABA neurons	10*ms*
*I*_*app*_	Applied current on DA and GABA neurons	varied

#### Modeling tonic synaptic currents

The transition from asynchronous synaptic inputs to tonic synaptic currents was done by reducing NMDAR, AMPAR and GABAR currents to the form of *I*_*NMDA*_ = *g*_*NMDA*_(*E*_*NMDA*_ − *v*), *I*_*AMPA*_ = *g*_*AMPA*_(*E*_*AMPA*_ − *v*) and $I_{GABA}={\bar{g}}_{GABA}(E_{GABA}-v)$ respectively. This corresponds to setting the receptor activation variables described by eq (13) to 1 to match dynamic clamp experiments [36,50]. Tonic receptor activation (*s* = 1) is a very good approximation of the asynchronous input (Compare Figs 1 and 2)

### Nullcline analysis

A multidimensional system can be analyzed with two-dimensional nullcline methods only after its reduction to a two-dimensional system. The description of this method is provided by Strogatz [104] and Rinzel and Ermentrout [29]. The reduction was done by eliminating the spike-producing currents, which do not significantly change the firing frequency of the neuron [4]. Nullclines are the curves where either $\frac{dv}{dt}=0$ or $\frac{d[Ca^{2+}]}{dt}=0$. Accordingly, nullclines of our system were obtained by numerically solving the following equations:

$$\begin{array}{l} \frac{dv}{dt}=\frac{1}{c_{m}}g_{Ca}(v)(E_{Ca}-v)+(g_{KCa}([Ca^{2+}])+g_{K}(v))(E_{K}-v)+g_{sNa}(v)(E_{Na}-v)+g_{l}(E_{l}-v)=0;\\ \frac{d[Ca^{2+}]}{dt}=\frac{2\beta }{r}\left(\frac{\left(g_{Ca}(v)+0.1g_{l}\right)}{zF}(E_{Ca}-v)-P_{Ca}[Ca^{2+}]\right)=0. \end{array}$$ (16)

The type of bifurcation was determined by systematically varying parameters of the system (in our case *g*_*GABA*_) until the behavior of the system qualitatively changes.

### Model of DA release

The model of DA release is adopted from Wightman and Zimmerman (1990) [105] and is described by the following equation

$$\frac{d[DA]}{dt}={\left[DA\right]}_{\text{max}}\delta (t-t_{spike})-\frac{V_{\text{max}}[DA]}{K_{m}+[DA]}$$ (17)

The first term describes the release from spiking activity of the DA neuron. Dirac delta function *δ*(*t* − *t*_*spike*_) represents the release at time of a spike. Maximum amount of DA released per spike is [*DA*]_max_ = 0.1*μM*. The second term represents DA uptake described by Michaelis-Menten equation, where *V*_max_ = 0.004*μM*/*ms* is the maximal rate of uptake by a transporter and *K*_*m*_ = 0.2*μM* is the affinity of the transporter for dopamine.

### Modeling a heterogeneous population of DA neurons and their inputs

Heterogeneity in the population of DA neurons was putatively introduced by varying the leak conductance. Further, neurons received correlated fluctuating NMDA inputs. NMDAR conductance to each DA neuron was given by a linear summation of Ornstein-Uhlenbeck (OU) processes [106] described as following:

$$g_{NMDA}(t)=\mu +\sigma (\sqrt{1-c}x_{i}(t)+\sqrt{c}x_{c}(t))$$ (18)

where *μ* = 1.5*mS*/*cm*^2^ and *σ* = 0.5*mS*/*cm*^2^ are the values of the mean and the standard deviation of the NMDAR conductance used for the simulations. *x*_*c*_(*t*) is the common component of the NMDA input that was applied to all of the DA neurons, whereas *x*_*i*_(*t*) is the independent component, which was generated individually for each neuron. A shared fraction of the input is determined by the input correlation *c* and was set to *c* = 0.5. Each OU process was formed by the following equation:

$$dx=-\frac{x}{\tau }dt+N_{\tau }x\sqrt{dt}$$ (19)

where *x*(*t*) is Gaussian white noise with zero mean and unit variance. *N*_*τ*_ = (2/*τ*)^1/2^ is a normalization constant that makes *x*(*t*) have unit variance. A correlation time *τ* = 5*ms* was used [33,64].

### Spike detection and firing pattern quantification in the model

In the model with fast sodium and the delayed rectifier potassium spike-producing currents, a spike was registered whenever voltage oscillation reached the threshold of 0 mV. In the reduced model (without spike-producing currents), a spike was registered every time voltage oscillations crossed the threshold of -40 mV, as experimentally it was shown that a DA neuron action potential is triggered when the voltage is depolarized to approximately -40 mV [3]. Voltage oscillations that were below these thresholds in the models with and without the spike-producing currents respectively were not counted as spikes and did not contribute to the firing frequency. To analyze firing pattern of simulated DA neuron in the presence of different synaptic currents, we quantified its firing rate and bursting. Mean firing rate of the simulated DA neuron was calculated as an inverse of the mean interspike interval (ISI). To calculate bursting we used ISI coefficient of variation (CV), calculated as the SD/mean of 200 ISIs.

## Supporting Information

S1 Text Comparison of the negative applied current and GABAR current influences on the DA neuron firing. (DOCX)

S1 Fig Smooth transition to zero frequency during application of hyperpolarizing current suggests type I excitability of the DA neuron. (A) The heat plot of the frequency distribution on the plane of NMDAR conductance and hyperpolarizing applied current for the model of the DA neuron. Note that the vertical axis shows the absolute value of the current. The transition to zero frequency remains smooth, which indicates type I excitability. However, the transition becomes steeper at greater hyperpolarizing applied currents. The insert illustrates a smooth frequency decrease during application of the negative current. (B 1–2) Two examples of the voltage traces corresponding to the parameters shown by red and black crosses in panel A. (B3) The rest state is formed at lower voltages in case of negative applied current (red bar) than in case of applied GABAR conductance (magenta bar). The value of NMDAR conductance is the same for both cases and indicated by a red cross in panel (A).
(EPS)

S2 Fig Comparison of the model behavior with and without spike producing currents (fast Na^2+^ current and delayed rectifier K^+^ current). A) Membrane potential waveform of a DA neuron model before (black line) and after (blue line) the fast Na current is blocked. B) A bifurcation diagram of the full model (with spike-producing currents) illustrates a SNIC bifurcation scenario. C) A smooth frequency decrease in the F-I curve of the full model corresponding to type I excitability. D) Same as in B) but for the reduced model (without spike-producing currents). E) Same as in C) but for the reduced model. The full model exhibits type I excitability as well as the reduced model.
(EPS)

S3 Fig AMPAR activation induces depolarization block in the DA neuron model with spike-producing currents. A) Dependence of the DA neuron firing frequency on the GABAR and AMPAR conductances. Firing persists in a much smaller range of GABAR conductance when GABAR is co-activated with AMPAR, similarly to the reduced model. B) Co-application of AMPA and GABA conductances induces depolarization block in a DA neuron model with spike-producing currents as well as in a reduced model.
(EPS)

## References

[1] Grace AA, Bunney BS. The control of firing pattern in nigral dopamine neurons: burst firing. J Neurosci. 1984;4: 2877–2890. Available: http://www.ncbi.nlm.nih.gov/pubmed/6150071 6150071 10.1523/JNEUROSCI.04-11-02877.1984 PMC6564720

[2] Hyland BI, Reynolds JN, Hay J, Perk CG, Miller R. Firing modes of midbrain dopamine cells in the freely moving rat. Neuroscience. 2002;114: 475–492. Available: http://www.ncbi.nlm.nih.gov/pubmed/12204216 12204216 10.1016/s0306-4522(02)00267-1

[3] Grace AA, Bunney BS. The control of firing pattern in nigral dopamine neurons: single spike firing. J Neurosci. 1984;4: 2866–2876. Available: http://www.ncbi.nlm.nih.gov/pubmed/6150070 6150070 10.1523/JNEUROSCI.04-11-02866.1984 PMC6564731

[4] Wilson CJ, Callaway JC. Coupled oscillator model of the dopaminergic neuron of the substantia nigra. J Neurophysiol. 2000;83: 3084–3100. 10805703 10.1152/jn.2000.83.5.3084

[5] Grace AA. Phasic versus tonic dopamine release and the modulation of dopamine system responsivity: a hypothesis for the etiology of schizophrenia. Neuroscience. 1991;41: 1–24. Available: http://www.ncbi.nlm.nih.gov/pubmed/1676137 1676137 10.1016/0306-4522(91)90196-u

[6] Nestler EJ, Carlezon WA Jr.. The mesolimbic dopamine reward circuit in depression. Biol Psychiatry. 2006;59: 1151–1159. 10.1016/j.biopsych.2005.09.018 16566899

[7] Grace AA, Onn SP. Morphology and electrophysiological properties of immunocytochemically identified rat dopamine neurons recorded in vitro. J Neurosci. 1989;9: 3463–3481. Available: http://www.ncbi.nlm.nih.gov/pubmed/2795134 2795134 10.1523/JNEUROSCI.09-10-03463.1989 PMC6569889

[8] Fujimura K, Matsuda Y. Autogenous oscillatory potentials in neurons of the guinea pig substantia nigra pars compacta in vitro. Neurosci Lett. 1989;104: 53–7. Available: http://www.ncbi.nlm.nih.gov/pubmed/2812536 2812536 10.1016/0304-3940(89)90328-5

[9] Yung WH, Häusser MA, Jack JJ. Electrophysiology of dopaminergic and non—dopaminergic neurones of the guinea—pig substantia nigra pars compacta in vitro. The Journal of Physiology 436 (1), 643–66, 1991 Available: https://www.ncbi.nlm.nih.gov/pubmed/2061849 2061849 10.1113/jphysiol.1991.sp018571 PMC1181526

[10] Kang Y, Kitai ST. A whole cell patch-clamp study on the pacemaker potential in dopaminergic neurons of rat substantia nigra compacta. Neurosci Res. 1993;18: 209–21. 8127469 10.1016/0168-0102(93)90056-v

[11] Shepard PD, Bunney BS. Repetitive firing properties of putative dopamine-containing neurons in vitro: regulation by an apamin-sensitive Ca2+-activated K+ conductance. Exp Brain Res. 1991;86: 141–150. 1756785 10.1007/BF00231048

[12] Mercuri NB, Bonci A, Calabresi P, Stratta F, Stefani A, Bernardi G. Effects of dihydropyridine calcium antagonists on rat midbrain dopaminergic neurones. Br J Pharmacol. 1994;113: 831–8. Available: http://www.pubmedcentral.nih.gov/articlerender.fcgi?artid=1510432&tool=pmcentrez&rendertype=abstract 7858874 10.1111/j.1476-5381.1994.tb17068.x PMC1510432

[13] Nedergaard S, Flatman JA, Engberg I. Nifedipine- and omega-conotoxin-sensitive Ca2+ conductances in guinea-pig substantia nigra pars compacta neurones. J Physiol. 1993;466: 727–747. 8410714 PMC1175500

[14] Wolfart J, Roeper J. Selective Coupling of T-Type Calcium Channels to SK Potassium Channels Prevents Intrinsic Bursting in Dopaminergic Midbrain Neurons. J Neurosci. 2002;22: 3404–3413. doi: 20026345 11978817 10.1523/JNEUROSCI.22-09-03404.2002 PMC6758365

[15] Wolfart J, Neuhoff H, Franz O, Roeper J. Differential expression of the small-conductance, calcium-activated potassium channel SK3 is critical for pacemaker control in dopaminergic midbrain neurons. J Neurosci. 2001;21: 3443–3456. 11331374 10.1523/JNEUROSCI.21-10-03443.2001 PMC6762487

[16] Harris NC, Webb C, Greenfield SA. A possible pacemaker mechanism in pars compacta neurons of the guinea-pig substantia nigra revealed by various ion channel blocking agents. Neuroscience. 1989;31: 355–362. 2552348 10.1016/0306-4522(89)90379-5

[17] Kang Y, Kitai ST. Calcium spike underlying rhythmic firing in dopaminergic neurons of the rat substantia nigra. Neurosci Res. 1993;18: 195–207. 7907413 10.1016/0168-0102(93)90055-u

[18] Kita T, Kita H, Kitai ST. Electrical membrane properties of rat substantia nigra compacta neurons in an in vitro slice preparation. Brain Res. 1986;372: 21–30. 3708356 10.1016/0006-8993(86)91454-x

[19] Chan CS, Guzman JN, Ilijic E, Mercer JN, Rick C, Tkatch T, et al “Rejuvenation” protects neurons in mouse models of Parkinson’s disease. Nature. 2007;447: 1081–1086. 10.1038/nature05865 17558391

[20] Amini B, Clark JW, Canavier CC. Calcium dynamics underlying pacemaker-like and burst firing oscillations in midbrain dopaminergic neurons: a computational study. J Neurophysiol. 1999;82: 2249–2261. 10561403 10.1152/jn.1999.82.5.2249

[21] Drion G, Massotte L, Sepulchre R, Seutin V. How modeling can reconcile apparently discrepant experimental results: The case of pacemaking in dopaminergic neurons. PLoS Comput Biol. 2011;7. 10.1371/journal.pcbi.1002050 PMC3102759 21637742

[22] Canavier CC, Landry RS. An increase in AMPA and a decrease in SK conductance increase burst firing by different mechanisms in a model of a dopamine neuron in vivo. J Neurophysiol. 2006;96: 2549–2563. 10.1152/jn.00704.2006 16885519 PMC2531289

[23] Komendantov AO, Komendantova OG, Johnson SW, Canavier CC. A modeling study suggests complementary roles for GABAA and NMDA receptors and the SK channel in regulating the firing pattern in midbrain dopamine neurons. J Neurophysiol. 2004;91: 346–357. 10.1152/jn.00062.2003 13679411

[24] Kuznetsov AS, Kopell NJ, Wilson CJ. Transient high-frequency firing in a coupled-oscillator model of the mesencephalic dopaminergic neuron. J Neurophysiol. 2006;95: 932–947. 10.1152/jn.00691.2004 16207783

[25] Puopolo M, Raviola E, Bean BP. Roles of subthreshold calcium current and sodium current in spontaneous firing of mouse midbrain dopamine neurons. J Neurosci. 2007;27: 645–656. 10.1523/JNEUROSCI.4341-06.2007 17234596 PMC6672803

[26] Guzman JN, Sánchez-Padilla J, Chan CS, Surmeier DJ. Robust pacemaking in substantia nigra dopaminergic neurons. J Neurosci. 2009;29: 11011–11019. 10.1523/JNEUROSCI.2519-09.2009 19726659 PMC2784968

[27] Khaliq ZM, Bean BP. Pacemaking in dopaminergic ventral tegmental area neurons: depolarizing drive from background and voltage-dependent sodium conductances. J Neurosci. 2010;30: 7401–7413. 10.1523/JNEUROSCI.0143-10.2010 20505107 PMC2892804

[28] Hodgkin AL. The local electric changes associated with repetitive action in a non-medullated axon. J Physiol. 1948;107: 165–181. Available: http://www.ncbi.nlm.nih.gov/pubmed/16991796 16991796 10.1113/jphysiol.1948.sp004260 PMC1392160

[29] Rinzel J, Ermentrout GB. Analysis of neural excitability and oscillations [Internet] Methods in neuronal modeling. 1989 pp. 251–292. http://mitpress.mit.edu/books/methods-neuronal-modeling

[30] Gutkin BS, Ermentrout GB. Dynamics of membrane excitability determine interspike interval variability: a link between spike generation mechanisms and cortical spike train statistics. Neural Comput. 1998;10: 1047–1065. 9654767 10.1162/089976698300017331

[31] Tateno T, Robinson HPC. Rate coding and spike-time variability in cortical neurons with two types of threshold dynamics. J Neurophysiol. 2006;95: 2650–2663. 10.1152/jn.00683.2005 16551842

[32] Jeong HY, Gutkin B. Synchrony of neuronal oscillations controlled by GABAergic reversal potentials. Neural Comput. 2007;19: 706–729. 10.1162/neco.2007.19.3.706 17298230

[33] Hong S, Ratte S, Prescott SA, De Schutter E. Single Neuron Firing Properties Impact Correlation-Based Population Coding. J Neurosci. 2012;32: 1413–1428. 10.1523/JNEUROSCI.3735-11.2012 22279226 PMC3571732

[34] Richards CD, Shiroyama T, Kitai ST. Electrophysiological and immunocytochemical characterization of GABA and dopamine neurons in the substantia nigra of the rat. Neuroscience. 1997;80: 545–557. Available: http://www.ncbi.nlm.nih.gov/pubmed/9284356 9284356 10.1016/s0306-4522(97)00093-6

[35] Lobb CJ, Wilson CJ, Paladini C a. A dynamic role for GABA receptors on the firing pattern of midbrain dopaminergic neurons. J Neurophysiol. 2010;104: 403–413. 10.1152/jn.00204.2010 20445035 PMC2904231

[36] Lobb CJ, Wilson CJ, Paladini C a. High-frequency, short-latency disinhibition bursting of midbrain dopaminergic neurons. J Neurophysiol. 2011;105: 2501–2511. 10.1152/jn.01076.2010 21367999 PMC3094190

[37] Chergui K, Charlety PJ, Akaoka H, Saunier CF, Brunet JL, Buda M, et al Tonic activation of NMDA receptors causes spontaneous burst discharge of rat midbrain dopamine neurons in vivo. Eur J Neurosci. 1993;5: 137–144. Available: http://www.ncbi.nlm.nih.gov/pubmed/8261095 8261095 10.1111/j.1460-9568.1993.tb00479.x

[38] Overton PG, Clark D. Burst firing in midbrain dopaminergic neurons. Brain Res Brain Res Rev. 1997;25: 312–334. Available: http://www.ncbi.nlm.nih.gov/pubmed/9495561 9495561 10.1016/s0165-0173(97)00039-8

[39] Morikawa H, Khodakhah K, Williams JT. Two intracellular pathways mediate metabotropic glutamate receptor-induced Ca2+ mobilization in dopamine neurons. J Neurosci. 2003;23: 149–157. Available: http://www.ncbi.nlm.nih.gov/pubmed/12514211 12514211 10.1523/JNEUROSCI.23-01-00149.2003 PMC1408315

[40] Deister CA, Teagarden MA, Wilson CJ, Paladini CA. An intrinsic neuronal oscillator underlies dopaminergic neuron bursting. J Neurosci. 2009;29: 15888–15897. 10.1523/JNEUROSCI.4053-09.2009 20016105 PMC2824818

[41] Tateno T. Threshold Firing Frequency-Current Relationships of Neurons in Rat Somatosensory Cortex: Type 1 and Type 2 Dynamics. J Neurophysiol. 2004;92: 2283–2294. 10.1152/jn.00109.2004 15381746

[42] Prescott SA, Ratte S, De Koninck Y, Sejnowski TJ. Pyramidal neurons switch from integrators in vitro to resonators under in vivo-like conditions. J Neurophysiol. 2008;100: 3030–3042. 10.1152/jn.90634.2008 18829848 PMC2604842

[43] Steriade M. Impact of network activities on neuronal properties in corticothalamic systems. J Neurophysiol. 2001;86: 1–39. 11431485 10.1152/jn.2001.86.1.1

[44] Franci A, Drion G, Seutin V, Sepulchre R. A Balance Equation Determines a Switch in Neuronal Excitability. PLoS Comput Biol. 2013;9. 10.1371/journal.pcbi.1003040 PMC3662658 23717194

[45] Drion G, Franci A, Seutin V, Sepulchre R. A novel phase portrait for neuronal excitability. PLoS One. 2012;7: 1–14. 10.1371/journal.pone.0041806 PMC3414513 22905107

[46] Putzier I, Kullmann PH, Horn JP, Levitan ES. Cav1.3 channel voltage dependence, not Ca2+ selectivity, drives pacemaker activity and amplifies bursts in nigral dopamine neurons. J Neurosci. 2009;29: 15414–15419. 10.1523/JNEUROSCI.4742-09.2009 20007466 PMC2796195

[47] Celada P, Paladini CA, Tepper JM. GABAergic control of rat substantia nigra dopaminergic neurons: Role of globus pallidus and substantia nigra pars reticulata. Neuroscience. 1999;89: 813–825. 10199615 10.1016/s0306-4522(98)00356-x

[48] Tepper JM, Lee CR. GABAergic control of substantia nigra dopaminergic neurons. Prog Brain Res. 2007;160: 189–208. 10.1016/S0079-6123(06)60011-3 17499115

[49] Lobb CJ, Wilson CJ, Paladini CA. High-frequency, short-latency disinhibition bursting of midbrain dopaminergic neurons. J Neurophysiol. 2011;105: 2501–2511. 10.1152/jn.01076.2010 21367999 PMC3094190

[50] Lobb CJ, Wilson CJ, Paladini CA. A dynamic role for GABA receptors on the firing pattern of midbrain dopaminergic neurons. J Neurophysiol. 2010;104: 403–413. 10.1152/jn.00204.2010 20445035 PMC2904231

[51] Wilson CJ, Callaway JC. Coupled oscillator model of the dopaminergic neuron of the substantia nigra. J Neurophysiol. 2000;83: 3084–3100. Available: http://www.ncbi.nlm.nih.gov/pubmed/10805703 10805703 10.1152/jn.2000.83.5.3084

[52] Izhikevich EM. Dynamical Systems in Neuroscience [Internet]. Dynamical Systems. 2007.

[53] Tateno T, Robinson HP. The mechanism of ethanol action on midbrain dopaminergic neuron firing: a dynamic-clamp study of the role of I(h) and GABAergic synaptic integration. J Neurophysiol. 2011;106: 1901–1922. 10.1152/jn.00162.2011 21697445

[54] McDaid J, McElvain MA, Brodie MS. Ethanol effects on dopaminergic ventral tegmental area neurons during block of Ih: involvement of barium-sensitive potassium currents. J Neurophysiol. 2008;100: 1202–10. 10.1152/jn.00994.2007 18614756 PMC2544469

[55] Johnson SW, North RA. Two types of neurone in the rat ventral tegmental area and their synaptic inputs. J Physiol. 1992;450: 455–468. Available: http://www.ncbi.nlm.nih.gov/pubmed/1331427 1331427 10.1113/jphysiol.1992.sp019136 PMC1176131

[56] Ha J, Kuznetsov A. Interaction of NMDA receptor and pacemaking mechanisms in the midbrain dopaminergic neuron. PLoS One. 2013;8: e69984 10.1371/journal.pone.0069984 23894569 PMC3716766

[57] Zakharov D, Lapish C, Gutkin B, Kuznetsov A. Synergy of AMPA and NMDA Receptor Currents in Dopaminergic Neurons: A Modeling Study. 2016;10: 1–11. 10.3389/fncom.2016.00048 PMC4877376 27252643

[58] Qian K, Yu N, Tucker KR, Levitan ES, Canavier CC. Mathematical analysis of depolarization block mediated by slow inactivation of fast sodium channels in midbrain dopamine neurons. J Neurophysiol. 2014;112: 2779–90. 10.1152/jn.00578.2014 25185810 PMC4254877

[59] Canavier CC. Sodium Dynamics Underlying Burst Firing and Putative Mechanisms for the Regulation of the Firing Pattern in Midbrain Dopamine Neurons: A Computational Approach. 1999;69: 49–69. 10.1023/a:1008809000182 10193646

[60] Lammel S, Hetzel A, Hackel O, Jones I, Liss B, Roeper J. Unique properties of mesoprefrontal neurons within a dual mesocorticolimbic dopamine system. Neuron. 2008;57: 760–773. 10.1016/j.neuron.2008.01.022 18341995

[61] Blythe SN, Wokosin D, Atherton JF, Bevan MD. Cellular mechanisms underlying burst firing in substantia nigra dopamine neurons. J Neurosci. 2009;29: 15531–15541. 10.1523/JNEUROSCI.2961-09.2009 20007477 PMC2834564

[62] Roeper J. Dissecting the diversity of midbrain dopamine neurons. Trends Neurosci. Elsevier Ltd; 2013;36: 336–342. 10.1016/j.tins.2013.03.003 23582338

[63] Ratté S, Hong S, DeSchutter E, Prescott SA. Impact of neuronal properties on network coding: Roles of spike initiation dynamics and robust synchrony transfer. Neuron. 2013;78: 758–772. 10.1016/j.neuron.2013.05.030 23764282 PMC3753823

[64] de la Rocha J, Doiron B, Shea-Brown E, Josić K, Reyes A. Correlation between neural spike trains increases with firing rate. Nature. 2007;448: 802–806. 10.1038/nature06028 17700699

[65] Ermentrout B. Type I membranes, phase resetting curves, and synchrony. Neural Comput. 1996;8: 979–1001. 8697231 10.1162/neco.1996.8.5.979

[66] Floresco SB, West AR, Ash B, Moore H, Grace A a. Afferent modulation of dopamine neuron firing differentially regulates tonic and phasic dopamine transmission. Nat Neurosci. 2003;6: 968–973. 10.1038/nn1103 12897785

[67] Paladini CA, Roeper J. Generating bursts (and pauses) in the dopamine midbrain neurons. Neuroscience. 2014;282C: 109–121. 10.1016/j.neuroscience.2014.07.032 25073045

[68] Anzalone A, Lizardi-Ortiz JE, Ramos M, De Mei C, Hopf FW, Iaccarino C, et al Dual control of dopamine synthesis and release by presynaptic and postsynaptic dopamine D2 receptors. J Neurosci. 2012;32: 9023–34. 10.1523/JNEUROSCI.0918-12.2012 22745501 PMC3752062

[69] Hu H, Vervaeke K, Storm JF. Two forms of electrical resonance at theta frequencies, generated by M-current, h-current and persistent Na+ current in rat hippocampal pyramidal cells. J Physiol. 2002;545: 783–805. 10.1113/jphysiol.2002.029249 12482886 PMC2290731

[70] Lobb CJ, Troyer TW, Wilson CJ, Paladini CA. Disinhibition bursting of dopaminergic neurons. Front Syst Neurosci. 2011;5: 25 10.3389/fnsys.2011.00025 21617731 PMC3095811

[71] Blythe SN, Atherton JF, Bevan MD. Synaptic activation of dendritic AMPA and NMDA receptors generates transient high-frequency firing in substantia nigra dopamine neurons in vitro. J Neurophysiol. 2007;97: 2837–2850. 10.1152/jn.01157.2006 17251363

[72] Yuan WX, He WS, Xue W-N, Wang Y, He S-M, Wang X-L, et al SK- and h-current contribute to the generation of theta-like resonance of rat substantia nigra pars compacta dopaminergic neurons at hyperpolarized membrane potentials. Brain Struct Funct. 2012;217: 379–94. 10.1007/s00429-011-0361-6 22108680

[73] Hutcheon B, Miura RM, Puil E. Models of subthreshold membrane resonance in neocortical neurons. J Neurophysiol. 1996;76: 698–714. 8871192 10.1152/jn.1996.76.2.698

[74] Wang W-T, Wan Y-H, Zhu J-L, Lei G-S, Wang Y-Y, Zhang P, et al Theta-frequency membrane resonance and its ionic mechanisms in rat subicular pyramidal neurons. Neuroscience. 2006;140: 45–55. 10.1016/j.neuroscience.2006.01.033 16527421

[75] Gastrein P, Campanac E, Gasselin C, Cudmore RH, Bialowas A, Carlier E, et al The role of hyperpolarization-activated cationic current in spike-time precision and intrinsic resonance in cortical neurons in vitro. J Physiol. 2011;589: 3753–73. 10.1113/jphysiol.2011.209148 21624967 PMC3171884

[76] Sacré P, Sepulchre R. Sensitivity analysis of oscillator models in the space of phase-response curves: Oscillators as open systems. 2012; http://arxiv.org/abs/1206.4144

[77] Tateno T, Robinson HPC. Phase resetting curves and oscillatory stability in interneurons of rat somatosensory cortex. Biophys J. Elsevier; 2007;92: 683–695. 10.1529/biophysj.106.088021 PMC1751383 17192317

[78] Gutkin B, Ermentrout GB. Spike Generating Dynamics and the Conditions for Spike-Time. 2003; 91–103. 10.1023/a:1024426903582 12843697

[79] St-Hilaire M, Longtin A. Comparison of coding capabilities of Type I and Type II neurons. J Comput Neurosci. 2004;16: 299–313. 10.1023/B:JCNS.0000025690.02886.93 15114051

[80] Prescott SA, Sejnowski TJ. Spike-Rate Coding and Spike-Time Coding Are Affected Oppositely by Different Adaptation Mechanisms. J Neurosci. 2008;28: 13649–13661. 10.1523/JNEUROSCI.1792-08.2008 19074038 PMC2819463

[81] Eshel N, Bukwich M, Rao V, Hemmelder V, Tian J, Uchida N. Arithmetic and local circuitry underlying dopamine prediction errors. Nature. Nature Publishing Group, a division of Macmillan Publishers Limited. All Rights Reserved.; 2015;525: 243–246. 10.1038/nature14855 PMC4567485 26322583

[82] Eshel N, Tian J, Bukwich M, Uchida N. Dopamine neurons share common response function for reward prediction error. Nat Neurosci. Nature Publishing Group; 2016; 1–11. 10.1038/nn.4239 PMC4767554 26854803

[83] Tobler PN. Adaptive Coding of Reward Value by Dopamine Neurons. Science (80-). 2005;307: 1642–1645. 10.1126/science.1105370 15761155

[84] Gutkin BS, Ermentrout GB, Reyes AD, S B, Phase-AR. Transient Inputs. J Neurophysiol. 2005; 1623–1635. 10.1152/jn.00359.2004 15829595

[85] Galan RF, Bard Ermentrout G, Urban NN. Reliability and stochastic synchronization in type I vs. type II neural oscillators. Neurocomputing. 2007;70: 2102–2106.

[86] Robinson DL, Howard EC, McConnell S, Gonzales RA, Wightman RM. Disparity between tonic and phasic ethanol-induced dopamine increases in the nucleus accumbens of rats. Alcohol Clin Exp Res. 2009;33: 1187–1196. 10.1111/j.1530-0277.2009.00942.x 19389195 PMC2947861

[87] Brodie MS, Appel SB. The effects of ethanol on dopaminergic neurons of the ventral tegmental area studied with intracellular recording in brain slices. Alcohol Clin Exp Res. 1998;22: 236–244. Available: http://www.ncbi.nlm.nih.gov/pubmed/9514313 9514313

[88] Okamoto T, Harnett MT, Morikawa H. Hyperpolarization-activated cation current (Ih) is an ethanol target in midbrain dopamine neurons of mice. J Neurophysiol. 2006;95: 619–626. 10.1152/jn.00682.2005 16148268 PMC1454360

[89] Saal D, Dong Y, Bonci A, Malenka RC. Drugs of abuse and stress trigger a common synaptic adaptation in dopamine neurons. Neuron. 2003;37: 577–582. 12597856 10.1016/s0896-6273(03)00021-7

[90] Theile JW, Morikawa H, Gonzales RA, Morrisett RA. Ethanol enhances GABAergic transmission onto dopamine neurons in the ventral tegmental area of the rat. Alcohol Clin Exp Res. 2008;32: 1040–1048. 10.1111/j.1530-0277.2008.00665.x 18422836 PMC2553853

[91] Morozova EO, Myroshnychenko M, Zakharov D, di Volo M, Gutkin BS, Lapish CC, Kuznetsov A. Contribution of synchronized GABAergic neurons to dopaminergic neuron firing and bursting. J Neurophysiol. 2016; jn.00232.2016. 10.1152/jn.00232.2016 PMC5144690 27440240

[92] Oster AM, Gutkin BS. A reduced model of DA neuronal dynamics that displays quiescence, tonic firing and bursting. J Physiol Paris. 2011;105: 53–58. 10.1016/j.jphysparis.2011.07.012 21939761

[93] Carter BC, Giessel AJ, Sabatini BL, Bean BP. Transient sodium current at subthreshold voltages: activation by EPSP waveforms. Neuron. 2012;75: 1081–1093. 10.1016/j.neuron.2012.08.033 22998875 PMC3460524

[94] Seutin V, Massotte L, Renette M-F, Dresse A. Evidence for a modulatory role of Ih on the firing of a subgroup of midbrain dopamine neurons. Neuroreport. 2001;12: 255 Available: http://journals.lww.com/neuroreport/Fulltext/2001/02120/Evidence_for_a_modulatory_role_of_Ih_on_the_firing.15.aspx/npapers2://publication/uuid/72AAD151-B52A-4217-A4CF-5B1F22D84861 11209930 10.1097/00001756-200102120-00015

[95] Li YX, Bertram R, Rinzel J. Modeling N-methyl-D-aspartate-induced bursting in dopamine neurons. Neuroscience. 1996;71: 397–410. Available: http://www.ncbi.nlm.nih.gov/pubmed/9053795 9053795 10.1016/0306-4522(95)00483-1

[96] Nair-Roberts RG, Chatelain-Badie SD, Benson E, White-Cooper H, Bolam JP, Ungless MA. Stereological estimates of dopaminergic, GABAergic and glutamatergic neurons in the ventral tegmental area, substantia nigra and retrorubral field in the rat. Neuroscience. 2008;152: 1024–1031. 10.1016/j.neuroscience.2008.01.046 18355970 PMC2575227

[97] Omelchenko N, Sesack SR. Ultrastructural analysis of local collaterals of rat ventral tegmental area neurons: GABA phenotype and synapses onto dopamine and GABA cells. Synapse. 2009;63: 895–906. 10.1002/syn.20668 19582784 PMC2741309

[98] Bourdy R, Barrot M. A new control center for dopaminergic systems: pulling the VTA by the tail. Trends Neurosci. 2012;35: 681–690. 10.1016/j.tins.2012.06.007 22824232

[99] van Zessen R, Phillips JL, Budygin EA, Stuber GD. Activation of VTA GABA neurons disrupts reward consumption. Neuron. 2012;73: 1184–1194. 10.1016/j.neuron.2012.02.016 22445345 PMC3314244

[100] Tepper JM, Martin LP, Anderson DR. GABAA receptor-mediated inhibition of rat substantia nigra dopaminergic neurons by pars reticulata projection neurons. J Neurosci. 1995;15: 3092–103. Available: http://www.ncbi.nlm.nih.gov/pubmed/7722648 7722648 10.1523/JNEUROSCI.15-04-03092.1995 PMC6577766

[101] Wang XJ, Buzsaki G. Gamma oscillation by synaptic inhibition in a hippocampal interneuronal network model. J Neurosci. 1996;16: 6402–6413. Available: http://www.ncbi.nlm.nih.gov/pubmed/8815919 8815919 10.1523/JNEUROSCI.16-20-06402.1996 PMC6578902

[102] Steffensen SC, Svingos AL, Pickel VM, Henriksen SJ. Electrophysiological characterization of GABAergic neurons in the ventral tegmental area. J Neurosci. 1998;18: 8003–8015. Available: http://www.ncbi.nlm.nih.gov/pubmed/9742167 9742167 10.1523/JNEUROSCI.18-19-08003.1998 PMC6793009

[103] Margolis EB, Toy B, Himmels P, Morales M, Fields HL. Identification of rat ventral tegmental area GABAergic neurons. PLoS One. 2012;7. 10.1371/journal.pone.0042365 PMC3409171 22860119

[104] Strogatz SH (Steven H. Nonlinear dynamics and chaos: with applications to physics, biology, chemistry, and engineering.

[105] Wightman RM, Zimmerman JB. Control of dopamine extracellular concentration in rat striatum by impulse flow and uptake. Brain Res Brain Res Rev. 1990;15: 135–144. Available: http://www.ncbi.nlm.nih.gov/pubmed/2282449 2282449 10.1016/0165-0173(90)90015-g

[106] Uhlenbeck GE, Ornstein LS. On the theory of the Brownian motion. Phys Rev. 1930;36: 823–841.

