# Supplementary material for: Dopamine Neurons Change the Type of Excitability in Response to Stimuli
Source: PLoS Comput Biol. 2016 Dec 8;12(12):e1005233. doi: 10.1371/journal.pcbi.1005233 (PMC5145155; doi:10.1371/journal.pcbi.1005233)
Supplement: S1 Text — (DOCX) [file pcbi.1005233.s001.docx]

**Supporting information**

To show that DA neuron exhibits type I excitability by standard definition of excitability type, we plot the frequency of the DA neuron as a function of the negative applied current (Fig. S1. A inset) instead of the GABAR conductance (as in Fig. 2 A 1 inset). We can see that indeed our DA neuron model is type I under the standard definition with a continuous F-I curve. Further, we extend the dependence into a 2-dimensional heat plot (Fig. S1. A main), where vertical axis is the hyperpolarizing current and the horizontal axis is NMDAR conductance as in Fig. 2 B. As expected from the dependence on the GABAR conductance (Fig. 2 A 1 inset), the frequency smoothly decreases to zero as a stronger hyperpolarizing current (negative) is injected. The similarity reflects that the increase of GABAR conductance increases the voltage-independent current given by $g_{GABA}E_{GABA}$ in (9), which is negative and equivalent to a hyperpolarizing injected current. Interestingly, the frequency dependence on hyperpolarizing current becomes steeper and the transition becomes more abrupt at higher NMDAR conductance, and the slope of the boundary increases (Fig. S1. A). Due to the greater slope of the boundary, the firing region narrows as the hyperpolarizing injected current grows, and the high-frequency firing can no longer be achieved. This is different from the results shown in Fig. 2 B because, as the GABAR conductance increases, the ohmic part, $g_{GABA}v$, induces a significant difference between the synaptic and injected currents. It is interesting to note that the slope of the boundary increases not due to a change in the type of excitability, but because the rest state under a hyperpolarizing current injection is formed at lower voltages (Fig. S.1 B3), where the NMDAR conductance (10) shuts off due to its magnesium block. By contrast, in the case of GABAR input (Fig. 2), the rest state cannot emerge at voltages that are below the GABAR reversal potential. Thus, we predict that DA neurons display the properties of type I excitability in control and in the balanced state.
